# Comprehensive overview of different medicinal parts from *Morus alba* L.: chemical compositions and pharmacological activities

**DOI:** 10.3389/fphar.2024.1364948

**Published:** 2024-04-17

**Authors:** Yumei Wang, Qing Ai, Meiling Gu, Hong Guan, Wenqin Yang, Meng Zhang, Jialin Mao, Zhao Lin, Qi Liu, Jicheng Liu

**Affiliations:** ^1^ The Research Institute of Medicine and Pharmacy, Qiqihar Medical University, Qiqihar, China; ^2^ School of Pharmacy, Qiqihar Medical University, Qiqihar, China; ^3^ Office of Academic Research, Qiqihar Medical University, Qiqihar, China

**Keywords:** *Mori folium*, *Mori ramulus*, *Mori cortex*, *Mori fructus*, chemical constituents, pharmacological activities

## Abstract

*Morus alba* L., a common traditional Chinese medicine (TCM) with a centuries-old medicinal history, owned various medicinal parts like Mori folium, Mori ramulus, Mori cortex and Mori fructus. Different medical parts exhibit distinct modern pharmacological effects. Mori folium exhibited analgesic, anti-inflammatory, hypoglycemic action and lipid-regulation effects. Mori ramulus owned anti-bacterial, anti-asthmatic and diuretic activities. Mori cortex showed counteraction action of pain, inflammatory, bacterial, and platelet aggregation. Mori fructus could decompose fat, lower blood lipids and prevent vascular sclerosis. The main chemical components in *Morus alba* L. covered flavonoids, phenolic compounds, alkaloids, and amino acids. This article comprehensively analyzed the recent literature related to chemical components and pharmacological actions of *M. alba* L., summarizing 198 of ingredients and described the modern activities of different extracts and the bioactive constituents in the four parts from *M. alba* L. These results fully demonstrated the medicinal value of *M. alba* L., provided valuable references for further comprehensive development, and layed the foundation for the utilization of *M. alba* L.

## 1 Introduction

*Morus alba* L., a deciduous tree species, belonging to the Moraceae family and *Morus* genus. China is the relatively early known country raising silkworms and growing *M. alba* L. The presence of *M. alba* L. could be traced back to thousands of years ago (Zeng et al., 2022). Besides, many medical classics such as *Shennong Ben Cao*, *Tang Ben Cao* and *Ben Cao Gang Mu also* recorded it (Wenmin Du, 2022). For the past few years, dozens of varieties of *M. alba* L. were widely planted in China, including cultivated and wild species (Ai et al., 2021). In TCM, *M. alba* L. is regarded as a treasure due to the rich active ingredients and modern activities of its different parts.

Mori cortex and Mori fructus taste slight cool and sweet. Mori cortex could purge and promoting water, relieve cough and asthma, reduce blood pressure, and against inflammatory (Batiha et al., 2023). Mori fructus could nourish blood and enhance immune function. Mori ramulus, which is mild and taste a litter bitter, owned functions of dispelling wind dampness and promoting blood circulation. Mori folium is a slight muted and possessed effects of dispelling wind, clearing heat, cooling blood, and improving eyesight. Mori fructus and Mori folium exhibit both medicinal and edible properties, making them widely used in medicine and food fields (Maqsood et al., 2022). In some Asian countries, Mori folium is used as a nutritional supplement (Liu et al., 2024). In South Korea, it is widely used as one ingredient of ice cream (Polumackanycz et al., 2021). In Japan, it is used as an anti-hyperglycemic supplement for the treatment of diabetes (Suthamwong et al., 2020). Recently, with the deepening awareness of *M. alba* L., its role in lowering blood sugar, alleviating depression, antioxidant and liver protection have been widely concerned.

The current pharmacological researches on *M. alba* L. mainly focus on Mori folium, Mori ramulus, Mori cortex and Mori fructus. With the rapid advance of science and technology, more bioactive substances covered flavonoids, alkaloids and phenols from *M. alba* L. were identified. In addition, there were several same biological active ingredients and some unique chemical components from different parts of *M. alba* L., the compositions were closely relevant to the pharmacological activities of each part. For example, 1-deoxynojirimycin, an alkaloid component only found in *M. alba* L., was the characteristic component with high-content from Mori folium, owns the intense inhibitory effect on α-glucosidase and exhibit obvious action in lowering blood glucose (Wang Shirui, 2023). Besides, on account of other affluent ingredients like proteins, carbohydrates, vitamins, trace elements and dietary fibre, Mori folium was also recognized as a high-quality food or mulberry tea (Polumackanycz et al., 2021). Thus it could be seen that due to the multifarious functional materials and particular pharmacological characteristics, different parts of *M. alba* L. maybe owned broad research prospects and were widely used in various scopes like medicine, food, and other fields (Maqsood et al., 2022).

On account of the favourable value of *M. alba* L., this review aimed to summarize the chemical components and the pharmacologic bioactivities of *M. alba* L., including Mori folium, Mori ramulus, Mori cortex and Mori fructus. The overall data in this present paper, could provide a helpful reference for further development and comprehensive utilization of *M. alba* L.

## 2 Chemical profiles of *Morus alba* L

Up to 198 active compounds have been identified in the different parts of *M. alba* L. (Supplementary Table S1; Tables 1–7). Their structures are summarized in Figures 1–8.

**TABLE 1:** Alkaloid in *Morus alba* L.

No.	Sequential number	Name	Source	Pharmacological properties	Reference
1	141	fagomine	B	anti-obesity; anti-inflammatory	D'Urso et al. (2019)
					Ramos-Romero et al. (2022)
2	142	morusimic acid B	B	__	D'Urso et al. (2019)
3	143	morusimic acid C	B	__	D'Urso et al. (2019)
4	144	morusimic acid E	B	__	D'Urso et al. (2019)
5	145	1-deoxynojirimycin	A; B	antidiabetic	D'Urso et al. (2019)
					Asai et al. (2011)
6	146	1,4-dideoxy-1,4-imino-D-arabinitol	D	hyperamnesia	Lei et al. (2022)
					Gibbs (2016)
7	147	2-formyl-1H-pyrrole-1-butanoic acid	B	__	D'Urso et al. (2019)
8	148	3-epi-fagomine	A	anticancer; neuroprotection	Amezqueta et al. (2012)
					Zabady et al. (2022)
					Bhuiyan et al. (2011)

Note: A, Mori folium; B, Mori fructus; C, Mori cortex; D, Mori ramulus.

**TABLE 2:** Coumarins in *Morus alba* L.

No.	Sequential number	Name	Source	Pharmacological property	Reference
1	149	aesculetin	A	anti-inflammatory	Li et al. (2020)
2	150	coumarin	C	anticancer; anti-inflammatory	Kavitha and Geetha (2018)
					Bhattarai et al. (2021)
3	151	mulberroside B	C	anti-obesity	Yang et al. (2011)
4	152	scopoletin	A	anti-inflammatory	Li et al. (2020)
5	153	scopolin	D	anti-inflammatory; anti-hyperuricemic	Yao et al. (2019)
					Li et al. (2020)
6	154	skimmin	A	cardioprotection	Doi et al. (2001)
					Su et al. (2023)
7	155	umbelliferone	C	antidiabetic nephropathy	Hyun et al. (2021)
					Jin and Chen (2022)
8	156	5,7-dihydroxycoumarin 7-O-β-d-apiofuranosyl-(1→6)-O-β-d-glucopyranoside	C	anti-obesity	Yang et al. (2011)
9	157	5,7-dihydroxycoumarin 7-O-β-D-glucopyranoside	C	anti-obesity	Yang et al. (2011)

Note: A, Mori folium; B, Mori fructus; C, Mori cortex; D, Mori ramulus.

**TABLE 3:** Carbohydrates in *Morus alba* L.

No.	Sequential number	Name	Source	Pharmacological property	Reference
1	158	adenosine	C	anti-obesity	Yang et al. (2011)
2	159	arabinose	A	anti-obesity	Zhao et al. (2022)
3	160	D-galactose	A	anti-obesity	Zhao et al. (2022)
4	161	D-galacturonic acid	A	anti-obesity	Zhao et al. (2022)
5	162	D-glucose	A	anti-obesity	Zhao et al. (2022)
6	163	D-glucuronic acid	A	anti-obesity	Zhao et al. (2022)
7	164	D-mannose	A	anti-obesity	Zhao et al. (2022)
8	165	fucose	A	anti-obesity	Zhao et al. (2022)
9	166	L-rhamnose	A	anti-obesity	Zhao et al. (2022)

Note: A, Mori folium; B, Mori fructus; C, Mori cortex; D, Mori ramulus.

**TABLE 4:** Terpenoids in *Morus alba* L.

No.	Sequential number	Name	Source	Pharmacological property	Reference
1	167	betulinic acid	C	anticancer; anti-obesity	Yang et al. (2011)
					Aswathy et al. (2022)
2	168	grasshopper ketone	B	anti-inflammatory	Lee et al. (2021b)
3	169	lanosterol acetate	A	antigout	Oh et al. (2021)
4	170	loliolide	A	antidiabetic; anti-inflammatory; anti-aging	Hunyadi et al. (2013)
					Park et al. (2019)
5	171	roseoside	B	anti-inflammatory	D'Urso et al. (2019)
					Wang et al. (2023b)
6	172	ursolic acid	C; D	anti-inflammatory; antioxidant; antiviral	Yang et al. (2011)
					Liu Ying (2023)
					Al-Kuraishy et al. (2022)
					Kornel et al. (2023)
7	173	uvaol	C	anti-obesity	Yang et al. (2011)
				anticancer	Bonel-Perez et al. (2020)
8	174	7-ketositosterol	B	kidney protection	Lee et al. (2021b)
9	175	β-sitosterol	C	anti-obesity; anticancer	Yang et al. (2011)
					Khan et al. (2022)

Note: A, Mori folium; B, Mori fructus; C, Mori cortex; D, Mori ramulus.

**TABLE 5:** Organic acids in *Morus alba* L.

No.	Sequential number	Name	Source	Pharmacological property	Reference
1	176	Acetic acid	A	skin protectant	Chen et al. (2021)
2	177	citric acid	A	immuno-enhancement	Chen et al. (2021)
					Hu et al. (2024)
3	178	fumaric acid	A	anticancer	Chen et al. (2021)
					Das et al. (2016)
4	179	lactic acid	A	anti-inflammatory; anticancer	Chen et al. (2021)
					Zhou et al. (2022a)
5	180	malic acid	A	antioxidant; liver protection	Chen et al. (2021)
					Koriem and Tharwat (2023)
6	181	succinic acid	A	anticancer	Chen et al. (2021)
					Kasarci et al. (2021)

Note: A, Mori folium; B, Mori fructus; C, Mori cortex; D, Mori ramulus.

**TABLE 6:** Anthocyanins in *Morus alba* L.

No.	Sequential number	Name	Source	Pharmacological property	Reference
1	182	anthocyanins	B	antioxidant; antibacterial	D'Urso et al. (2019)
					Suriyaprom et al. (2021)
2	183	cyanidin-3-glucoside	B	anticancer	Zabady et al. (2022)
3	184	cyanidin-3-O-glucoside	B	anticancer	Chen et al. (2022c)
					Wei et al. (2021)
4	185	cyanidin-3-O-rutinoside	B	antioxidant	Chen et al. (2022d)
					Delazar et al. (2010)

Note: A, Mori folium; B, Mori fructus; C, Mori cortex; D, Mori ramulus.

**TABLE 7:** Other constituents in *Morus alba* L.

No.	Sequential number	Name	Category	Source	Pharmacological property	Reference
1	186	butyl pyroglutamate	amino acid derivatives	B	kidney protection	Lee et al. (2018)
2	187	γ-aminobutyric acid	amino acids	A	antifatigue	Chen et al. (2016)
3	188	L-proline	amino acids	B	anti-inflammatory; kidney protection	Lee et al. (2021b)
						Li et al. (2019)
4	189	L-tryptophan	amino acids	A	antipyretic; mood improvement; sleep improvement	Qu et al. (2019)
						Sutanto et al. (2022)
						Kikuchi et al. (2021)
5	190	chalcomoracin	Diels–Alder adducts	A	anti-bacteria	Jeon and Choi (2019)
						Kim et al. (2012)
6	191	guangsangon E	Diels–Alder adducts	A	anticancer	Shu et al. (2021)
7	192	isobavachalcone	chalcones	B	antidiabetic; antioxidant; anti-inflammatory; neuroprotection; antimicrobial	Wang et al. (2013)
						Wang et al. (2021)
8	193	morachalcone A	chalcones	D	anti-melanogenesis	Zhang et al. (2016)
9	194	2,4,2′,4′-tetrahydroxychalcone	chalcones	D	anti-melanogenesis	Zhang et al. (2016)
10	195	lignin	phenylpropanoids	A	anti-microbial	Chao et al. (2021)
						Das et al. (2024)
11	196	melatonin	amines	A	antioxidant; anticancer; anti-aging	Panyatip et al. (2022)
						Bhattacharya et al. (2019)
12	197	vitamin E	vitamins	C	antioxidant; anti-inflammatory	Kavitha and Geetha (2018)
13	198	cyclo (L-Pro-L-Val)	peptides	B	anti-inflammatory	Lee et al. (2021b)

Note: A, Mori folium; B, Mori fructus; C, Mori cortex; D, Mori ramulus.

**FIGURE 1:**
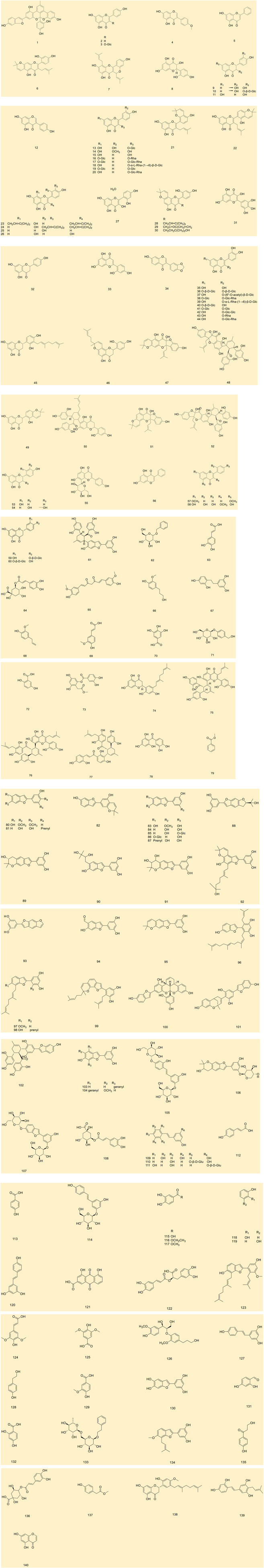
Phenols.

**FIGURE 2:**
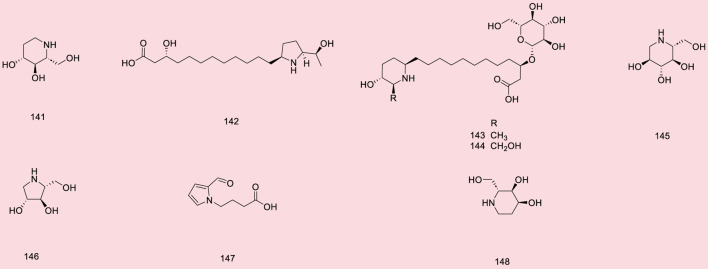
Alkaloids.

**FIGURE 3:**
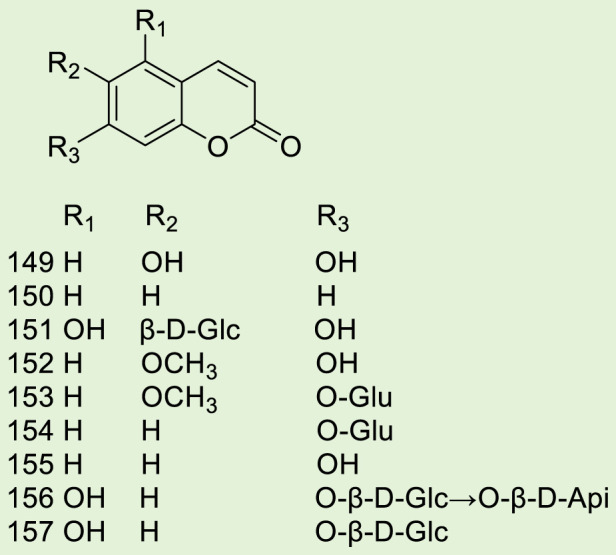
Coumarins.

**FIGURE 4:**
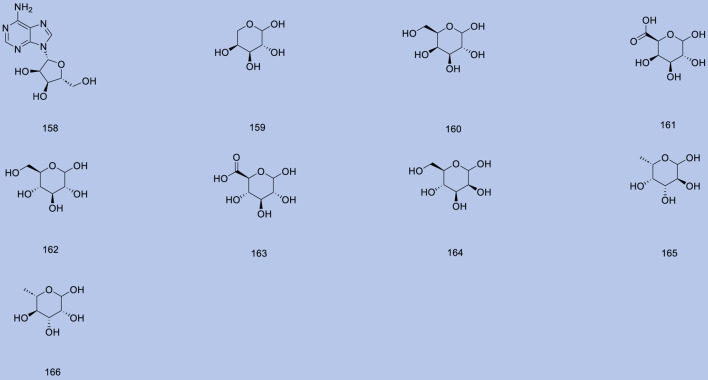
Carbohydrates.

**FIGURE 5:**
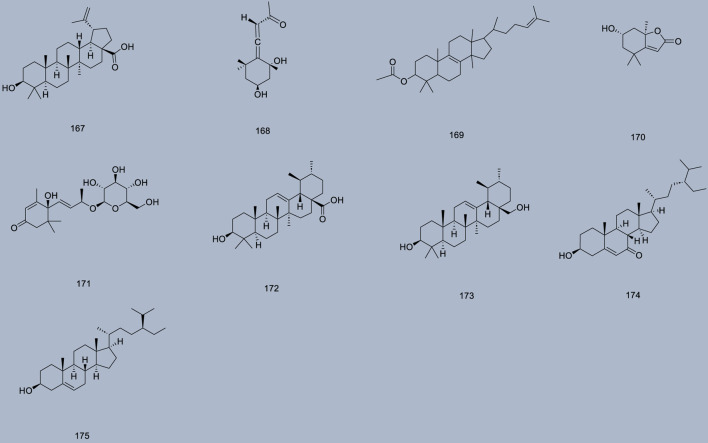
Terpenoids.

**FIGURE 6:**
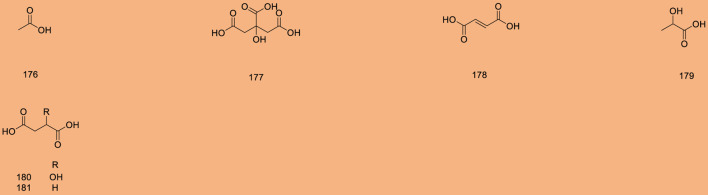
Organic acids.

**FIGURE 7:**
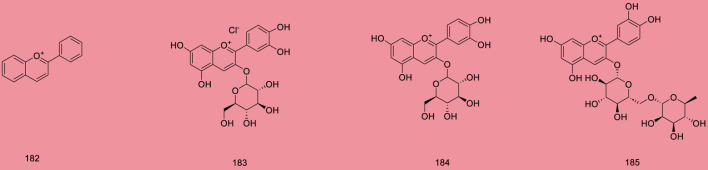
Anthocyanin.

**FIGURE 8:**
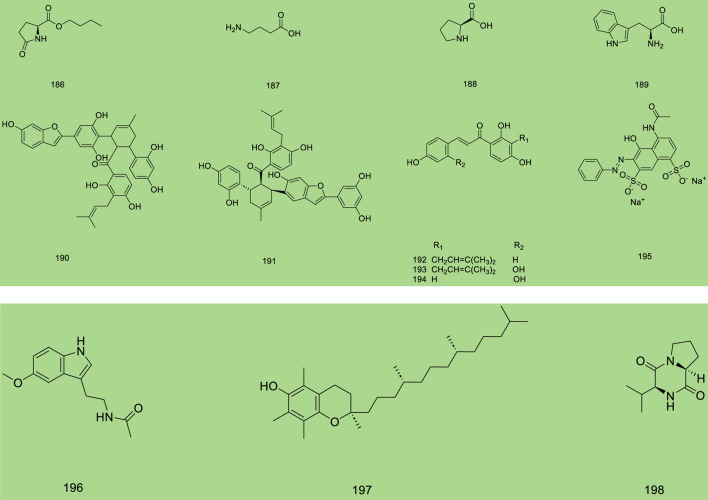
Other categories.

## 3 The pharmacological activities of components in *Morus alba* L

### 3.1 Hypoglycemic activity

1-deoxynojirimycin was the important active ingredient in *M. alba* L. Researchers have confirmed that 1-deoxynojirimycin exhibit an inhibitory effect on α-glucosidase, further reduced the postprandial blood glucose in pre-diabetic and mildly diabetic individuals (Asai et al., 2011). Current evidence showed that the same dose of Mori folium has similar biological activities like lowering blood sugar and protecting kidney in diabetic patient as the purified 1-deoxynojirimycin (Huang et al., 2014). In addition, Mori ramulus extract was reported effective hypoglycemic action and well inhibition of PTP1B and α-glucosidase, the main components were oxyresveratrol and kuwanon G (Kwon et al., 2022). Compared with Mori folium and Mori fructus, the hypoglycemic effects of Mori ramulus and Mori cortex were much more significant (Zhou Q. Y. et al., 2022). The various bioactive components of medicinal parts from *M. alba* L. expressed multiple antidiabetic targets and less adverse reactions. Thanks to the favourable hypoglycemic effect and the accessibility of *M. alba* L. resources, *M. alba* L. may exhibit a promising prospect in the preventing and treating of diabetes. The main hypoglycemic compounds in *M. alba* L. and their mechanisms are shown in Table 8.

**TABLE 8:** Hypoglycemic mechanisms of components from *Morus alba* L.

Mechanism	Component	Reference
inhibition of α-glucosidase	chalcomoracin	Liu et al. (2022)
	chlorogenic acid; rutin	Hunyadi et al. (2012)
	dihydromorin; kuwanon C; kuwanon G; moracin M; norartocarpetin	Kwon et al. (2022)
	kuwanon H	Zhou et al. (2022b)
	morin	Przeor (2022)
	morusin; morusinol	Zhou et al. (2022b)
	oxyresveratrol	Kwon et al. (2022)
	1-deoxynojirimycin	Jan et al. (2022)
Enhancement of glucose uptake via in-sulin signaling pathway/AMP-activated protein kinase	isoquercetin	Lim et al. (2021)
Increase insulin secretion of pancreatic β-cells	syringic acid	Jan et al. (2022)

### 3.2 Antioxidant activity

Studies found that isoquercetin and 4-O-caffeoylquinic acid in Mori folium showed strong antioxidant activity, and the 50% radical-scavenging concentrations were 10.63 ± 0.96 μg/mL and 10.63 ± 0.96 μg/mL, respectively (Ganzon et al., 2018). The researchers comprehensively evaluated the antioxidant activities of bioactive components from *M. alba* L. in DPPH and ABTS radical scavenging assays, found that the acetone extract showed potential antioxidant activities with SC_50_ values of 242.33 ± 15.78 and 129.28 ± 10.53 μg/mL, respectively (Hsu et al., 2022). The antioxidant mechanisms of components from *M. alba* L. are summarized in Table 9.

**TABLE 9:** Antioxidant mechanisms of components from *Morus alba* L.

Mechanism	Component	Reference
inhibition of ROS production	astragalin; kaempferol; luteolin; quercetin; taxifolin	Yu et al. (2021)
inhibition of soluble epoxide hydrolase	aesculetin; moracin B; moracin J; moracin M; moracin M 3′-O-β-glucopyranoside; mulberroside F; scopoletin; scopoline	Li et al. (2020)
scavenging or inhibiting the production of free radicals	anthocyanins	Suriyaprom et al. (2021)
	caffeic acid; chlorogenic acid; ferulic acid; gallic acid; myricetin; naringenin; p-coumaric acid; rosmarinic acid; rutin; sinapinic acid	Polumackanycz et al. (2021)
	mulberroside A; oxyresveratrol	Thomas et al. (2022)
	protocatechuic acid; isoquercetin	Leyva-Jimenez et al. (2020)
	4-O-caffeoylquinic acid	Ganzon et al. (2018)

### 3.3 Anti-inflammatory activity

Studies have demonstrated that *M. alba* L. and its active compounds could inhibit the inflammation by suppressing leukocyte chemotaxis, further data about the mechanism showed that oxyresveratrol in *M. alba* L. could inhibit the CXCR4-mediated leukocyte migration of the CXCR4 receptor by inactivating the MEK/ERK pathway (Chen et al., 2013). In addition, oxyresveratrol was alos reported favourable anti-inflammatory effect through the inhibitions of iNOS/NO production, synthesis of PGE2 and activation of NF-κB(Chung et al., 2003). The methanol extraction of mulberry bark showed that components named kuwanon T and sanggenon A in mulberry bark contribute to the anti-inflammatory activities on microglia (BV2) and macrophages (RAW264.7) by the inhibitions of productions of prostaglandin E2, interleukin-6 and tumour necrosis factor-α, and the stimulation of expression of cyclooxygenase-2 (Ko et al., 2021). The anti-inflammatory active ingredients in mulbery are displayed in Table 10.

**TABLE 10:** Anti-inflammatory mechanisms of components from *Morus alba* L.

Mechanism	Name	Reference
inhibition the release of pro-inflammatory cytokines	mulberroside A	Shi et al. (2023)
	protocatechuic acid; isoquercetin	Leyva-Jimenez et al. (2020)
inhibiting MEK/ERK signaling in leukocyte migration	oxyresveratrol	Chen et al. (2013)
inhibition of NF-κB pathway activity	morusin	Jia et al. (2020)
	moracin O; moracin P	Hardianti et al. (2020)
	neochlorogenic acid	Gao et al. (2020a)
	kuwanon T	Ko et al. (2021)
	sanggenon A	
downregulating INOS expression	astragalin; kaempferol; luteolin; quercetin; taxifolin	Yu et al. (2021)
regulating Nrf2 signaling pathways	neochlorogenic acid	Gao et al. (2020b)
	kuwanon T	Ko et al. (2021)
	sanggenon A	
removal of excess reactive oxygen/nitrogen species or interaction with their interacting enzymes	cudraflavone B; kuwanon E; 4′-O-methylkuwanon E	Kollar et al. (2013)
selective inhibition of COX-2 activity	kuwanon A	Baek et al. (2021)

### 3.4 Anti-cancer activity

Moracin D was demonstrated that it could decrease cell proliferation and induce apoptosis in breast cancer cells by inhibiting the transduction pathway of Wnt3a/FOXM1/β-catenin signal and the activation of caspase and GSK3β(Hwang et al., 2018). Sanggenol L, another natural flavonoid compound in Mori cortex, could induce the apoptosis through inhibiting the PI3K/Akt/mTOR signaling pathway, and accelerate the cycle arrest of prostate cancer cells by activating the p53 protein (Won and Seo, 2020). In addition, sanggenol L could also reduce cytotoxicity and apoptosis in ovarian cancer cells through activating cysteine aspartase and inhibiting NF-κB (Ko et al., 2021). Moracin N was an active ingredient in Mori folium, which exhibit anti-lung cancer properties through apoptosis and autophagy (Gao C. et al., 2020). Morusin, which separate from Mori cortex, was demonstrated effective anticancer activity by inhibiting the vitality of prostate cancer cells with minimal impact on normal prostate epithelial cells, reducing STAT3 activity via the inhibition of phosphorylation, nuclear accumulation and DNA-binding activity. Moreover, morusin showed well downregulation effect on the expression of STAT1 target genes of Cyclin D2. Furthermore, morusin could also decrease the activity of STAT3 in inducing the apoptosis in prostate cancer cells (Lim et al., 2015). The anti-cancer components in *M. alba* L. and their mechanisms are summarized in Table 11.

**TABLE 11:** Anticancer mechanisms of components from *Morus alba* L.

Mechanism	Component	Reference
inhibition of the Akt/mTOR signalling pathway	morusin	Wu et al. (2023)
activating AMP-activated protein kinase	morusin	Park and Park (2020)
reducing STAT3 activity	morusin	Cho et al. (2017)
regulating bax and survivin expression	morusin	Kang et al. (2017)
induces autophagy	guangsangon E	Shu et al. (2021)
	moracin N	Gao et al. (2020b)
regulation of autophagy protein ATG3L16-related RNA molecule expression	cyanidin-3-glucoside	Zabady et al. (2022)
activating protein and inhibiting of signaling	sanggenol L	Won and Seo (2020)
	moracin D	Hwang et al. (2018)
inhibition of HIF-1α in tumours and DLL4 activity in the endothelium	steppogenin	Cha et al. (2023)
targeting the KDM4B-MYC axis	sanggenon C	Tang et al. (2023)
induces CHK1 degradation through the ubiquitin-proteasome pathway	morusinol	Guo et al. (2023)

### 3.5 Other activities

Beside the aforementioned activities, the ingredients in the different parts from *M. alba* L. also exhibit other activities such as melanin inhibition effect, hair growth, *etc*. Up to now, multiple constituents including norluciferin, moracin B, moracin J, moracin M-3′-O-β-glucopyranoside and moracin M-6-O-β-D-glucopyranoside againsting melanin were separated from the ethanol extracts of *M. alba* L. These components have a significant dose-dependent inhibition of melanin production, effectively suppressed the activity of tyrosinase in B10-F1 cells induced by α-melanocyte stimulating hormone and exhibited inhibitory effects on the expression of associated proteins, such as microphthalmia-associated transcription factor, tyrosinase, and tyrosinase-associated protein-1 (Li Y. et al., 2018). Mulberroside F in Mori folium exhibit inhibitory effect on melanin and through the inhibition of tyrosinase and the formation of melanin in melanin-A cells (Lee et al., 2002). Moreover, little Mori cortex extract showed the stimulating on hair growth, enhance the secretion of growth factors, facilitating the transition of hair follicles from the resting phase to the growth phase, activating β-linker proteins, which is essential for inducing the growth phase (Hyun et al., 2021). Other pharmacological activities and the related mechanisms are summarized in Table 12.

**TABLE 12:** Other pharmacological effects and their mechanisms of components from *Morus alba* L.

Activity	Mechanism	Component	Reference
antigout	blocking the RAS signaling pathway	lanosterol acetate	Oh et al. (2021)
antiviral	interference with cell damage caused by influenza virus infection	gallic acid	Kim and Chung (2018)
	direct inhibition of influenza virus entry	morin hydrate	Hong et al. (2020)
	inhibition of viral neuraminidase	sanggenon C	Langeder et al. (2023a)
	inhibition of SARS-CoV-2 proteases	sanggenon C; sanggenon G; sanggenon O	Wasilewicz et al. (2023)
antiulcer	inhibition the releasion of histamine	quercetin	Garg et al. (2022)
	inhibition the formation of platelet-activating factor	rutin	Garg et al. (2022)
antidepressant	interacts with the 5-hydroxytryptaminergic	sanggenon G	Lim et al. (2015)
antiplatelet	inhibition of thromboxane release	mulberroside C	Kwon et al. (2021)
	inhibition of platelet aggregation	morusinol	Lee et al. (2012)
anti-fatigue	increased glucose phosphatase activity	γ-aminobutyric	Chen et al. (2016)
anti-melanogenic	inhibition of tyrosinase activity	kuwanon G; mulberrofuran G	Koirala et al. (2018)
	inhibition of S1P lyase activity	mulberroside A; oxyresveratrol	Zheng et al. (2023)
anti-obesity	regulation of gut microbial communities and lipid indices	arabinose; D-galactose; D-galacturonic acid; D-glucose; D-glucuronic acid; D-mannose	Yang et al. (2022a)
		fucose	
		L-rhamnose	
anti-bacteria	blocking the binding of [1–^14^C]acetate to *Staphylococcus aureus* membrane lipids	chalcomoracin; moracin C	Kim et al. (2012)
neuroprotection	maintenance of mitochondrial membrane potential and mitochondrial function	cyanidin-3-glucoside	Bhuiyan et al. (2011)
	promoted nuclear translocation of the mitophagy regulator TFEB and activated the AMPK-ULK1 pathway	morin	Wang et al. (2023c)
cardioprotection	enhancement autophagy of hypoxia-induced	sanggenon C	Gu et al. (2017)
prevent hair loss	increased secretion of angiogenic paracrine factors	chlorogenic acid; umbelliferone	Hyun et al. (2021)
anti-alzheimer’s disease	reduction of intracellular amyloid-β oligomer-induced cytotoxicity	anthocyanins	Ochiishi et al. (2021)
against Benzo [a]pyrene in epidermal keratinocytes	activation of Nrf2-mediated signaling/inhibition of aryl hydrocarbon receptor signaling	maclurin	Kim et al. (2021)
relieve fever	inhibition of arachidonic acid metabolic pathway	tryptophan	Qu et al. (2019)
anti-hyperuricemia	inhibition of xanthine oxidase activity, and downregulation expression of mURAT1, mGLUT9, and mABCG2	polydatin	Ge et al. (2023)

## 4 Future perspective

In recent years, with the continuous advancement of modern science and technology, researchers conducted in-depth investigations of multifarious constituents and pharmacological activities of *M. alba* L., including Mori folium, Mori ramulus, Mori cortex and Mori fructus, making its high medicinal potential valuable in contemporary society. Until now, there were some reviews about the pharmacologic activities of *M. alba* L. (Hao et al., 2022), however, in view of the crucial connection between pharmacological actions and ingredients, the revelation of the overall constituents of *M. alba* L. was extremely important. When referred to constituents of *M. alba* L. concluded in this paper, the primary constituents were phenols, flavonoids, alkaloids, *etc.*. Summing up the pharmaceutical actions of *M. alba* L., hypoglycemic, antioxidant and anti-inflammatory were the common activities, and different constituents may owned similar effects. As is well konwn that, the connection between ingredients’s structure and pharmaceutical effects was extremely important. Take flavonoids ingredients, for example, the diversiform flavonoids in *M. alba* L. exhibited anti-inflammatory action. Popularly, the modifications could affect the mechanisms of inflammation, including glycosylation, hydroxylation, *etc.* (Chen et al., 2018). For example, both quercetin and its glycoside derivative quercetine-3-glucoside exhibit same anti-inflammatory activity with distinctive mechanisms of action. Quercetin downregulated the INOS expression (Leyva-Jimenez et al., 2020), however, isoquercetin inhibited the release of pro-inflammatory cytokines (Yu et al., 2021). Besides, different activities of constituents may owned the similar mechanisms. For example, AMP-activated protein kinase was related to both the anti-hpyerglycemic effect and anti-cancer action of *M. alba* L. Besides, when referred to the anti-oxidant and anti-inflammatory activities of *M. alba* L., the inhibition of soluble epoxide hydrolase was the same mechanism. These information indicated that one mechanism maybe related to diversified activities of *M. alba* L. based on the similar compounds. To sum up, the ingredients of *M. alba* L. were diverse, and the effect owned the characteristics of multiple approaches and multiple targets.

Nowadays, in order to extend the application of *M. alba L.* in TCM and food, the toxicity assessments of *M. alba* L. were evaluated by various experiments. When referred to Mori folium, the LD_50_ was higher than 15.0 g/kg bw in the acute toxicity test, indicating that Mori folium was deemed as safe and it may own a wide application as food or nutritional supplements (Li Y. et al., 2018). Besides, Mori fructus was a familiar edible food in daily life, and it was widely made into diverse foods such as fresh/dried fruit, fruit wine/juice, and other healthcare foods. From the sub-chronic oral toxicity test, the safe dose without observed adverse was up to 4200 mg/kg, meaning that Mori fructus was nontoxic under conventional edible dosage. Until now, there were none reports about the acute or chronic toxicity of the extracts of Mori cortex. However, the maximum tolerated dose of oral administration of the active ingredient named sanggenon C, an active ingredient derived from Mori cortex as well as identificated in Mori ramulus, was up to 100 mg/g (Langeder et al., 2023b). However, resveratrol, another active constituent in Mori cortex and Mori ramulus, was reported controversial toxicity, the metabolites of resveratrol may exhibit cytotoxic effects (Shaito et al., 2020), meaning that Mori cortex and Mori ramulus may owned a ralative reasonable safe space when applied. Hence, in order to improve the expansive value of *M. alba* L., the detailed illustrations of acute and long-term toxicity of Mori cortex and Mori ramulus were particularly vital in further studies for researchers.

## 5 Conclusion

This review summarized the chemical profiles and the pharmacological activities of *M. alba* L., as well as the safety and the structure-activity relationship. Totally 198 of constituents including phenols, alkaloids, coumarins, carbohydrates, terpenoids, organic acids, anthocyanins, and other constituents were concluded. Among the chemical ingredients, 140 of them were phenols, indicating that phenols may played a critical role in this plant. Modern pharmacological research showed that *M. alba* L. exhibited hypoglycemic, antioxidant, anti-inflammatory, anti-cancer and other activities, illustrating that *M. alba* L. has showed favourable applications in pharmaceutical and food fields. Furhter biological activities and the related mechanisms of the ingredients in *M. alba* L. were needed in order to promote the development of pharmaceutical industry. In addition, more nutritional valve analysis and toxicity research data were particularly important for the development of *M. alba* L. in food scope.

## References

[B1] Agus H. H. Cetin A. Ozdemir N. Ozbay M. G. Caglar M. A. Sariyildiz M. A. (2022). Resorcinol alleviates alpha-terpineol-induced cell death in *Schizosaccharomyces pombe* via increased activity of the antioxidant enzyme Sod2. FEMS Yeast Res. 22 (1), foac052. 10.1093/femsyr/foac052 36309474

[B2] Ai J. Bao B. Battino M. Giampieri F. Chen C. You L. (2021). Recent advances on bioactive polysaccharides from mulberry. Food Funct. 12 (12), 5219–5235. 10.1039/d1fo00682g 34019048

[B3] Alavi D. S. Farkhondeh T. Aschner M. Darroudi M. Samini H. Samarghandian S. (2023). Chrysin effect against gastric cancer: focus on its molecular mechanisms. Curr. Molec. Pharmacol. 16 (7), 707–711. 10.2174/1874467216666230103105725 36597606

[B4] Alibakhshi A. Malekzadeh R. Hosseini S. A. Yaghoobi H. (2023). Investigation of the therapeutic role of native plant compounds against colorectal cancer based on system biology and virtual screening. Sci. Rep. 13 (1), 11451. 10.1038/s41598-023-38134-5 37454152 PMC10349871

[B5] Al-Kuraishy H. M. Al-Gareeb A. I. Negm W. A. Alexiou A. Batiha G. E. (2022). Ursolic acid and SARS-CoV-2 infection: a new horizon and perspective. Inflammopharmacology 30 (5), 1493–1501. 10.1007/s10787-022-01038-3 35922738 PMC9362167

[B6] Amezqueta S. Galan E. Fuguet E. Carrascal M. Abian J. Torres J. L. (2012). Determination of D-fagomine in buckwheat and mulberry by cation exchange HPLC/ESI-Q-MS. Anal. Bioanal. Chem. 402 (5), 1953–1960. 10.1007/s00216-011-5639-2 22207282

[B7] Asai A. Nakagawa K. Higuchi O. Kimura T. Kojima Y. Kariya J. (2011). Effect of mulberry leaf extract with enriched 1-deoxynojirimycin content on postprandial glycemic control in subjects with impaired glucose metabolism. J. Diabetes Investig. 2 (4), 318–323. 10.1111/j.2040-1124.2011.00101.x PMC4014974 24843505

[B8] Aswathy M. Vijayan A. Daimary U. D. Girisa S. Radhakrishnan K. V. Kunnumakkara A. B. (2022). Betulinic acid: a natural promising anticancer drug, current situation, and future perspectives. J. Biochem. Mol. Toxicol. 36 (12), e23206. 10.1002/jbt.23206 36124371

[B9] Baek S. Hwang S. Park T. Kwon Y. Cho M. Park D. (2021). Evaluation of selective COX-2 inhibition and *in silico* study of kuwanon derivatives isolated from *Morus alba* . Int. J. Mol. Sci. 22 (7), 3659. 10.3390/ijms22073659 33915826 PMC8036738

[B10] Batiha G. E. Al-Snafi A. E. Thuwaini M. M. Teibo J. O. Shaheen H. M. Akomolafe A. P. (2023). *Morus alba*: a comprehensive phytochemical and pharmacological review. Schmiedeb. Arch. Pharmacol. 396 (7), 1399–1413. 10.1007/s00210-023-02434-4 PMC10244279 36877269

[B11] Bhattacharya S. Patel K. K. Dehari D. Agrawal A. K. Singh S. (2019). Melatonin and its ubiquitous anticancer effects. Mol. Cell. Biochem. 462 (1-2), 133–155. 10.1007/s11010-019-03617-5 31451998

[B12] Bhattarai N. Kumbhar A. A. Pokharel Y. R. Yadav P. N. (2021). Anticancer potential of coumarin and its derivatives. Mini-Rev. Med. Chem. 21 (19), 2996–3029. 10.2174/1389557521666210405160323 33820507

[B13] Bhuiyan M. I. H. Kim H. Kim S. Y. Cho K. (2011). The neuroprotective potential of cyanidin-3-glucoside fraction extracted from mulberry following oxygen-glucose deprivation. Korean J. Physiology Pharmacol. 15 (6), 353–361. 10.4196/kjpp.2011.15.6.353 PMC3282223 22359473

[B14] Bonel-Perez G. C. Perez-Jimenez A. Gris-Cardenas I. Parra-Perez A. M. Lupianez J. A. Reyes-Zurita F. J. (2020). Antiproliferative and pro-apoptotic effect of uvaol in human hepatocarcinoma HepG2 cells by affecting G0/G1 cell cycle arrest, ROS production and AKT/PI3K signaling pathway. Molecules 25 (18), 4254. 10.3390/molecules25184254 32947962 PMC7571068

[B15] Cha S. Kim H. Jang H. Lee J. Chao T. Baek N. (2023). Steppogenin suppresses tumor growth and sprouting angiogenesis through inhibition of HIF-1α in tumors and DLL4 activity in the endothelium. Phytomedicine 108, 154513. 10.1016/j.phymed.2022.154513 36332389

[B16] Chaita E. Lambrinidis G. Cheimonidi C. Agalou A. Beis D. Trougakos I. (2017). Anti-melanogenic properties of Greek plants. A novel depigmenting agent from *Morus alba* wood. Mol Basel, Switz. 22 (4), 514. 10.3390/molecules22040514 PMC6154579 28333105

[B17] Chao N. Yu T. Hou C. Liu L. Zhang L. (2021). Genome-wide analysis of the lignin toolbox for *morus* and the roles of lignin related genes in response to zinc stress. PeerJ (San Francisco, CA) 9, e11964. e11964-e11964. 10.7717/peerj.11964 PMC8351576 34434666

[B18] Chen C. Mohamad Razali U. H. Saikim F. H. Mahyudin A. Mohd Noor N. Q. I. (2021). *Morus alba* L. Plant: bioactive compounds and potential as a functional food ingredient. Foods 10 (3), 689. 10.3390/foods10030689 33807100 PMC8004891

[B19] Chen C. Mokhtar R. A. M. Sani M. S. A. Noor N. Q. I. M. (2022a). The effect of maturity and extraction solvents on bioactive compounds and antioxidant activity of mulberry (*Morus alba*) fruits and leaves. Molecules 27 (8), 2406. 10.3390/molecules27082406 35458604 PMC9029729

[B20] Chen H. He X. Liu Y. Li J. He Q. Zhang C. (2016). Extraction, purification and anti-fatigue activity of gamma-aminobutyric acid from mulberry (*Morus alba* L.) leaves. Sci. Rep. 6, 18933. 10.1038/srep18933 26743028 PMC4705516

[B21] Chen J. Li G. Sun C. Peng F. Yu L. Chen Y. (2022b). Chemistry, pharmacokinetics, pharmacological activities, and toxicity of Quercitrin. Phytother. Res. 36 (4), 1545–1575. 10.1002/ptr.7397 35253930

[B22] Chen L. Teng H. Xie Z. Cao H. Cheang W. S. Skalicka-Woniak K. (2018). Modifications of dietary flavonoids towards improved bioactivity: an update on structure-activity relationship. Crit. Rev. Food Sci. Nutr. 58 (4), 513–527. 10.1080/10408398.2016.1196334 27438892

[B23] Chen T. Shuang F. Fu Q. Ju Y. Zong C. Zhao W. (2022c). Evaluation of the chemical composition and antioxidant activity of mulberry (*Morus alba* L.) fruits from different varieties in China. Molecules 27 (9), 2688. 10.3390/molecules27092688 35566039 PMC9102544

[B24] Chen X. Han Y. Chen L. Tian Q. L. Yin Y. L. Zhou Q. (2022d). Discovery and characterization of the flavonoids in Cortex Mori Radicis as naturally occurring inhibitors against intestinal nitroreductases. Chem.-Biol. Interact. 368, 110222. 10.1016/j.cbi.2022.110222 36244406

[B25] Chen Y. C. Tien Y. J. Chen C. H. Beltran F. N. Amor E. C. Wang R. J. (2013). *Morus alba* and active compound oxyresveratrol exert anti-inflammatory activity via inhibition of leukocyte migration involving MEK/ERK signaling. BMC Complement. Altern. Med. 13, 45. 10.1186/1472-6882-13-45 23433072 PMC3639811

[B26] Cho S. W. Na W. Choi M. Kang S. J. Lee S. Choi C. Y. (2017). Autophagy inhibits cell death induced by the anti-cancer drug morusin. Am. J. Cancer Res. 7 (3), 518–530. 28401008 PMC5385640

[B27] Choi S. W. Jang Y. J. Lee Y. J. Leem H. H. Kim E. O. (2013). Analysis of functional constituents in mulberry (*Morus alba* L.) twigs by different cultivars, producing areas, and heat processings. Prev. Nutr. Food Sci. 18 (4), 256–262. 10.3746/pnf.2013.18.4.256 24551827 PMC3925215

[B28] Das A. K. Mitra K. Conte A. J. Sarker A. Chowdhury A. Ragauskas A. J. (2024). Lignin - a green material for antibacterial application - a review. Int. J. Biol. Macromol. 261 (Pt 2), 129753. 10.1016/j.ijbiomac.2024.129753 38286369

[B29] Das R. K. Brar S. K. Verma M. (2016). Recent advances in the biomedical applications of fumaric acid and its ester derivatives: the multifaceted alternative therapeutics. Pharmacol. Rep. 68 (2), 404–414. 10.1016/j.pharep.2015.10.007 26922546

[B30] Delazar A. Khodaie L. Afshar J. Nahar L. Sarker S. (2010). Isolation and free-radical-scavenging properties of cyanidin 3-O-glycosides from the fruits of Ribes biebersteinii Berl. Acta Pharm. Zagreb. Croat. 60 (1), 1–11. 10.2478/v10007-010-0007-x 20228037

[B31] Doi K. Kojima T. Makino M. Kimura Y. Fujimoto Y. (2001). Studies on the constituents of the leaves of *Morus alba* L. Chem. Pharm. Bull. 49 (2), 151–153. 10.1248/cpb.49.151 11217100

[B32] Dong H. Yu P. Long B. Peng T. He Y. Xu B. (2023). Total synthesis of kuwanons A and B and discovery of their antibacterial mechanism. J. Nat. Prod. 86 (8), 2022–2030. 10.1021/acs.jnatprod.3c00466 37499116

[B33] D'Urso G. Mes J. J. Montoro P. Hall R. D. de Vos R. C. H. (2019). Identification of bioactive phytochemicals in mulberries. Metabolites 10 (1), 7. 10.3390/metabo10010007 31861822 PMC7023076

[B34] Funakoshi-Tago M. Nonaka Y. Tago K. Takeda M. Ishihara Y. Sakai A. (2020). Pyrocatechol, a component of coffee, suppresses LPS-induced inflammatory responses by inhibiting NF-κB and activating Nrf2. Sci. Rep. 10 (1), 2584. 10.1038/s41598-020-59380-x 32054966 PMC7018815

[B35] Ganzon J. G. Chen L. G. Wang C. C. (2018). 4-O-Caffeoylquinic acid as an antioxidant marker for mulberry leaves rich in phenolic compounds. J. Food Drug Anal. 26 (3), 985–993. 10.1016/j.jfda.2017.11.011 29976416 PMC9303035

[B36] Gao C. Sun X. Wu Z. Yuan H. Han H. Huang H. (2020a). A novel benzofuran derivative moracin N induces autophagy and apoptosis through ROS generation in lung cancer. Front. Pharmacol. 11, 391. 10.3389/fphar.2020.00391 32477104 PMC7235196

[B37] Gao X. H. Zhang S. D. Wang L. T. Yu L. Zhao X. L. Ni H. Y. (2020b). Anti-inflammatory effects of neochlorogenic acid extract from mulberry leaf (*Morus alba* L.) against LPS-stimulated inflammatory response through mediating the AMPK/Nrf2 signaling pathway in A549 cells. Molecules 25 (6), 1385. 10.3390/molecules25061385 32197466 PMC7144357

[B38] Garg S. Singla R. K. Rahman M. M. Sharma R. Mittal V. (2022). Evaluation of ulcer protective activity of *Morus alba* L. Extract-loaded chitosan microspheres in ethanol-induced ulcer in rat model. Evid.-based Complement. Altern. Med. 2022, 4907585–4907617. 10.1155/2022/4907585 PMC9546716 36212972

[B39] Ge X. Su Z. Wang Y. Zhao X. Hou K. Zheng S. (2023). Identifying the intervention mechanisms of polydatin in hyperuricemia model rats by using UHPLC-Q-Exactive Orbitrap mass spectroscopy metabonomic approach. Front. Nutr. 10, 1117460. 10.3389/fnut.2023.1117460 37187876 PMC10176606

[B40] Gibbs M. E. (2016). Role of glycogenolysis in memory and learning: regulation by noradrenaline, serotonin and ATP. Neurosci 9, 70. 10.3389/fnint.2015.00070 PMC4717441 26834586

[B41] Grienke U. Richter M. Walther E. Hoffmann A. Kirchmair J. Makarov V. (2016). Discovery of prenylated flavonoids with dual activity against influenza virus and Streptococcus pneumoniae. Sci. Rep. 6 (1), 27156. 10.1038/srep27156 27257160 PMC4891693

[B42] Gu Y. Gao L. Chen Y. Xu Z. Yu K. Zhang D. (2017). Sanggenon C protects against cardiomyocyte hypoxia injury by increasing autophagy. Mol. Med. Rep. 16 (6), 8130–8136. 10.3892/mmr.2017.7646 28983604 PMC5779897

[B43] Guo L. Dong Z. Zhang X. Yang Y. Hu X. Ji Y. (2023). Morusinol extracted from *Morus alba* induces cell cycle arrest and apoptosis via inhibition of DNA damage response in melanoma by CHK1 degradation through the ubiquitin-proteasome pathway. Phytomedicine 114, 154765. 10.1016/j.phymed.2023.154765 37004403

[B44] Guzman L. Balada C. Flores G. Alvarez R. Knox M. Vinet R. (2018). t-Resveratrol protects against acute high glucose damage in endothelial cells. Plant Food Hum. Nutr. 73 (3), 235–240. 10.1007/s11130-018-0683-0 30039194

[B45] Hao J. Gao Y. Xue J. Yang Y. Yin J. Wu T. (2022). Phytochemicals, pharmacological effects and molecular mechanisms of mulberry. Foods 11 (8), 1170. 10.3390/foods11081170 35454757 PMC9028580

[B46] Hardianti B. Umeyama L. Li F. Yokoyama S. Hayakawa Y. (2020). Anti-inflammatory compounds moracin O and P from *Morus alba* Linn. (Sohakuhi) target the NF-kappaB pathway. Mol. Med. Rep. 22 (6), 5385–5391. 10.3892/mmr.2020.11615 33173971 PMC7647032

[B47] Hong E. Song J. Kim S. Cho J. Jeong B. Yang H. (2020). Morin hydrate inhibits influenza virus entry into host cells and has anti-inflammatory effect in influenza-infected mice. Immune Netw. 20 (4), e32. 10.4110/in.2020.20.e32 32895619 PMC7458794

[B48] Hsu J. H. Yang C. S. Chen J. J. (2022). Antioxidant, anti-alpha-glucosidase, antityrosinase, and anti-inflammatory activities of bioactive components from *Morus alba* . Antioxidants 11 (11), 2222. 10.3390/antiox11112222 36421408 PMC9686747

[B49] Hu P. Yuan M. Guo B. Lin J. Yan S. Huang H. (2024). Citric acid promotes immune function by modulating the intestinal barrier. Int. J. Mol. Sci. 25 (2), 1239. 10.3390/ijms25021239 38279237 PMC10817003

[B50] Hu X. Yu M. Yan G. Wang H. Hou A. Lei C. (2018). Isoprenylated phenolic compounds with tyrosinase inhibition from *Morus nigra* . J. Asian Nat. Prod. Res. 20 (5), 488–493. 10.1080/10286020.2017.1350653 29191050

[B51] Hua F. Li J. Y. Zhang M. Zhou P. Wang L. Ling T. J. (2022). Kaempferol-3-O-rutinoside exerts cardioprotective effects through NF-κB/NLRP3/Caspase-1 pathway in ventricular remodeling after acute myocardial infarction. J. Food Biochem. 46 (10), e14305. 10.1111/jfbc.14305 35758877

[B52] Huang S. S. Yan Y. H. Ko C. H. Chen K. M. Lee S. C. Liu C. T. (2014). A comparison of food-grade folium mori (sang ye) extract and 1-deoxynojirimycin for glycemic control and renal function in streptozotocin-induced diabetic rats. J. Tradit. Complement. Med. 4 (3), 162–170. 10.4103/2225-4110.131639 25161921 PMC4142454

[B53] Hunyadi A. Liktor-Busa E. Márki Á. Martins A. Jedlinszki N. Hsieh T. J. (2013). Metabolic effects of mulberry leaves: exploring potential benefits in type 2 diabetes and hyperuricemia. Evid.-based Complement. Altern. Med. 2013, 948627–948710. 10.1155/2013/948627 PMC3870074 24381639

[B54] Hunyadi A. Martins A. Hsieh T. J. Seres A. Zupko I. (2012). Chlorogenic acid and rutin play a major role in the *in vivo* anti-diabetic activity of *Morus alba* leaf extract on type II diabetic rats. PLoS One 7 (11), e50619. 10.1371/journal.pone.0050619 23185641 PMC3503931

[B55] Hwang S. M. Lee H. J. Jung J. H. Sim D. Y. Hwang J. Park J. E. (2018). Inhibition of wnt3a/FOXM1/β-catenin Axis and activation of GSK3β and caspases are critically involved in apoptotic effect of moracin D in breast cancers. Int. J. Mol. Sci. 19 (9), 2681. 10.3390/ijms19092681 30201862 PMC6164368

[B56] Hyun J. Im J. Kim S. Kim H. Y. Seo I. Bhang S. H. (2021). *Morus alba* root extract induces the anagen phase in the human hair follicle dermal papilla cells. Pharmaceutics 13 (8), 1155. 10.3390/pharmaceutics13081155 34452116 PMC8399394

[B57] Islam A. Islam M. S. Rahman M. K. Uddin M. N. Akanda M. R. (2020). The pharmacological and biological roles of eriodictyol. Arch. Pharm. Res. 43 (6), 582–592. 10.1007/s12272-020-01243-0 32594426

[B58] Jan B. Zahiruddin S. Basist P. Irfan M. Abass S. Ahmad S. (2022). Metabolomic profiling and identification of antioxidant and antidiabetic compounds from leaves of different varieties of *Morus alba* linn grown in kashmir. ACS Omega 7 (28), 24317–24328. 10.1021/acsomega.2c01623 35874221 PMC9301699

[B59] Jeon Y. Choi S. (2019). Isolation, identification, and quantification of tyrosinase and α-glucosidase inhibitors from UVC-irradiated mulberry (*Morus alba* L.) leaves. Prev. Nutr. Food Sci. 24 (1), 84–94. 10.3746/pnf.2019.24.1.84 31008101 PMC6456241

[B60] Jeong J. Y. Liu Q. Kim S. B. Jo Y. H. Mo E. J. Yang H. H. (2015). Characterization of melanogenesis inhibitory constituents of *Morus alba* leaves and optimization of extraction conditions using response surface methodology. Molecules 20 (5), 8730–8741. 10.3390/molecules20058730 26007176 PMC6272263

[B61] Jia Y. He W. Zhang H. He L. Wang Y. Zhang T. (2020). Morusin ameliorates IL-1β-induced chondrocyte inflammation and osteoarthritis via NF-κB signal pathway. Drug Des. Devel Ther. 14, 1227–1240. 10.2147/DDDT.S244462 PMC7105369 32273685

[B62] Jin T. Chen C. (2022). Umbelliferone delays the progression of diabetic nephropathy by inhibiting ferroptosis through activation of the Nrf-2/HO-1 pathway. Food Chem. Toxicol. 163, 112892. 10.1016/j.fct.2022.112892 35278496

[B63] Jongkon N. Seaho B. Tayana N. Prateeptongkum S. Duangdee N. Jaiyong P. (2022). Computational analysis and biological activities of oxyresveratrol analogues, the putative cyclooxygenase-2 inhibitors. Mol Basel, Switz. 27 (7), 2346. 10.3390/molecules27072346 PMC9000610 35408774

[B64] Ju W. T. Kwon O. C. Kim H. B. Sung G. B. Kim H. W. Kim Y. S. (2018). Qualitative and quantitative analysis of flavonoids from 12 species of Korean mulberry leaves. J. Food Sci. Technol.-Mysore. 55 (5), 1789–1796. 10.1007/s13197-018-3093-2 PMC5897299 29666531

[B65] Kang S. Kim E. Kim S. Lee J. Ahn K. S. Yun M. (2017). Morusin induces apoptosis by regulating expression of Bax and Survivin in human breast cancer cells. Oncol. Lett. 13 (6), 4558–4562. 10.3892/ol.2017.6006 28599457 PMC5453030

[B66] Kasarci G. Ertugrul B. Iplik E. S. Cakmakoglu B. (2021). The apoptotic efficacy of succinic acid on renal cancer cell lines. Med. Oncol. 38 (12), 144. 10.1007/s12032-021-01577-9 34687367

[B67] Kavitha Y. Geetha A. (2018). Anti-inflammatory and preventive activity of white mulberry root bark extract in an experimental model of pancreatitis. J. Tradit. Complement. Med. 8 (4), 497–505. 10.1016/j.jtcme.2018.01.011 30302330 PMC6174261

[B68] Khan Z. Nath N. Rauf A. Emran T. B. Mitra S. Islam F. (2022). Multifunctional roles and pharmacological potential of β-sitosterol: emerging evidence toward clinical applications. Chem.-Biol. Interact. 365, 110117. 10.1016/j.cbi.2022.110117 35995256

[B69] Kikuchi A. M. Tanabe A. Iwahori Y. (2021). A systematic review of the effect of L-tryptophan supplementation on mood and emotional functioning. J. Diet. Suppl. 18 (3), 316–333. 10.1080/19390211.2020.1746725 32272859

[B70] Kim D. Lee K. Kwon J. Lee H. J. Lee D. Mar W. (2015). Neuroprotection against 6-OHDA-induced oxidative stress and apoptosis in SH-SY5Y cells by 5,7-Dihydroxychromone: activation of the Nrf2/ARE pathway. Life Sci. 130, 25–30. 10.1016/j.lfs.2015.02.026 25818191

[B71] Kim D. S. Irfan M. Sung Y. Y. Kim S. H. Park S. H. Choi Y. H. (2017). Schisandra chinensis and *Morus alba* synergistically inhibit *in vivo* thrombus formation and platelet aggregation by impairing the glycoprotein VI pathway. Evid.-based Complement. Altern. Med. 2017, 7839658. 10.1155/2017/7839658 PMC5286545 28194217

[B72] Kim D. S. Kang Y. M. Jin W. Y. Sung Y. Y. Choi G. Kim H. K. (2014). Antioxidant activities and polyphenol content of *Morus alba* leaf extracts collected from varying regions. Biomed. Rep. 2 (5), 675–680. 10.3892/br.2014.294 25054010 PMC4106594

[B73] Kim H. Chung M. S. (2018). Antiviral activities of mulberry (*Morus alba*) juice and seed against influenza viruses. Evid.-based Complement. Altern. Med. 2018, 2606583. 10.1155/2018/2606583 PMC6236660 30515232

[B74] Kim J. Park S. Yang S. Oh S. W. Kwon K. Park S. J. (2021). Protective effects of maclurin against benzo[a]pyrene via aryl hydrocarbon receptor and nuclear factor erythroid 2-related factor 2 targeting. Antioxidants 10 (8), 1189. 10.3390/antiox10081189 34439437 PMC8388905

[B75] Kim J. H. Doh E. J. Lee G. (2020a). Quantitative comparison of the marker compounds in different medicinal parts of *Morus alba* L. Using high-performance liquid chromatography-diode array detector with chemometric analysis. Molecules 25 (23), 5592. 10.3390/molecules25235592 33261214 PMC7730820

[B76] Kim M. Nam D. Ju W. Choe J. Choi A. (2020b). Response surface methodology for optimization of process parameters and antioxidant properties of mulberry (*Morus alba* L.) leaves by extrusion. Molecules 25 (22), 5231. 10.3390/molecules25225231 33182637 PMC7697072

[B77] Kim Y. J. Sohn M. Kim W. Korea R. I. O. B. (2012). Chalcomoracin and moracin C, new inhibitors of *Staphylococcus aureus* enoyl-acyl carrier protein reductase from *Morus alba* . Biol. Pharm. Bull. 35 (5), 791–795. 10.1248/bpb.35.791 22687419

[B78] Ko W. Liu Z. Kim K. Dong L. Lee H. Kim N. Y. (2021). Kuwanon T and sanggenon a isolated from *Morus alba* exert anti-inflammatory effects by regulating NF-κB and HO-1/Nrf2 signaling pathways in BV2 and RAW264.7 cells. Molecules 26 (24), 7642. 10.3390/molecules26247642 34946724 PMC8708433

[B79] Koirala P. Seong S. H. Zhou Y. Shrestha S. Jung H. A. Choi J. S. (2018). Structure(-)Activity relationship of the tyrosinase inhibitors kuwanon G, mulberrofuran G, and albanol B from *morus* species: a kinetics and molecular docking study. Molecules 23 (6), 1413. 10.3390/molecules23061413 29891812 PMC6099663

[B80] Kollar P. Barta T. Hosek J. Soucek K. Zavalova V. M. Artinian S. (2013). Prenylated flavonoids from *Morus alba* L. Cause inhibition of G1/S transition in THP-1 human leukemia cells and prevent the lipopolysaccharide-induced inflammatory response. Evid.-based Complement. Altern. Med. 2013, 350519. 10.1155/2013/350519 PMC3671669 23762124

[B81] Koriem K. M. M. Tharwat H. A. K. (2023). Malic acid improves behavioral, biochemical, and molecular disturbances in the hypothalamus of stressed rats. J. Integr. Neurosci. 22 (4), 98. 10.31083/j.jin2204098 37519180

[B82] Kornel A. Nadile M. Retsidou M. I. Sakellakis M. Gioti K. Beloukas A. (2023). Ursolic acid against prostate and urogenital cancers: a review of *in vitro* and *in vivo* studies. Int. J. Mol. Sci. 24 (8), 7414. 10.3390/ijms24087414 37108576 PMC10138876

[B83] Kwon H. W. Lee D. H. Rhee M. H. Shin J. H. (2021). *In vitro* antiplatelet activity of mulberroside C through the up-regulation of cyclic nucleotide signaling pathways and down-regulation of phosphoproteins. Genes 12 (7), 1024. 10.3390/genes12071024 34209363 PMC8305937

[B84] Kwon R. H. Thaku N. Timalsina B. Park S. E. Choi J. S. Jung H. A. (2022). Inhibition mechanism of components isolated from *Morus alba* branches on diabetes and diabetic complications via experimental and molecular docking analyses. Antioxidants 11 (2), 383. 10.3390/antiox11020383 35204264 PMC8869400

[B85] Langeder J. Döring K. Schmietendorf H. Grienke U. Schmidtke M. Rollinger J. M. (2023a). 1H NMR-based biochemometric analysis of *Morus alba* extracts toward a multipotent herbal anti-infective. J. Nat. Prod. 86 (1), 8–17. 10.1021/acs.jnatprod.2c00481 36543521 PMC9887597

[B86] Langeder J. Koch M. Schmietendorf H. Tahir A. Grienke U. Rollinger J. M. (2023b). Correlation of bioactive marker compounds of an orally applied *Morus alba* root bark extract with toxicity and efficacy in BALB/c mice. Front. Pharmacol. 14, 1193118. 10.3389/fphar.2023.1193118 38143489 PMC10739329

[B87] Lee D. Lee S. R. Kang K. S. Kim K. H. (2021a). Bioactive phytochemicals from mulberry: potential anti-inflammatory effects in lipopolysaccharide-stimulated RAW 264.7 macrophages. Int. J. Mol. Sci. 22 (15), 8120. 10.3390/ijms22158120 34360887 PMC8348635

[B88] Lee D. Lee S. R. Park B. J. Song J. H. Kim J. K. Ko Y. (2021b). Identification of renoprotective phytosterols from mulberry (*Morus alba*) fruit against cisplatin-induced cytotoxicity in LLC-PK1 kidney cells. Plants 10 (11), 2481. 10.3390/plants10112481 34834844 PMC8623081

[B89] Lee D. Yu J. Lee S. Hwang G. Kang K. Park J. (2018). Beneficial effects of bioactive compounds in mulberry fruits against cisplatin-induced nephrotoxicity. Int. J. Mol. Sci. 19 (4), 1117. 10.3390/ijms19041117 29642519 PMC5979275

[B90] Lee J. J. Yang H. Yoo Y. M. Hong S. S. Lee D. Lee H. J. (2012). Morusinol extracted from *Morus alba* inhibits arterial thrombosis and modulates platelet activation for the treatment of cardiovascular disease. J. Atheroscler. Thromb. 19 (6), 516–522. 10.5551/jat.10058 22472211

[B91] Lee S. H. Choi S. Y. Kim H. Hwang J. S. Lee B. G. Gao J. J. (2002). Mulberroside F isolated from the leaves of *Morus alba* inhibits melanin biosynthesis. Biol. Pharm. Bull. 25 (8), 1045–1048. 10.1248/bpb.25.1045 12186407

[B92] Lee Y. J. Lee S. Y. (2021). Maclurin exerts anti-cancer effects in human osteosarcoma cells via prooxidative activity and modulations of PARP, p38, and ERK signaling. IUBMB Life 73 (8), 1060–1072. 10.1002/iub.2506 34003554

[B93] Lei L. Huan Y. Liu Q. Li C. Cao H. Ji W. (2022). *Morus alba* L. (Sangzhi) alkaloids promote insulin secretion, restore diabetic β-cell function by preventing dedifferentiation and apoptosis. Front. Pharmacol. 13, 841981. 10.3389/fphar.2022.841981 35308210 PMC8927674

[B94] Leyva-Jimenez F. J. Ruiz-Malagon A. J. Molina-Tijeras J. A. Diez-Echave P. Vezza T. Hidalgo-Garcia L. (2020). Comparative study of the antioxidant and anti-inflammatory effects of leaf extracts from four different *Morus alba* genotypes in high fat diet-induced obesity in mice. Antioxidants 9 (8), 733. 10.3390/antiox9080733 32796677 PMC7465205

[B95] Li H. Li S. Yang H. Wang Y. Wang J. Zheng N. (2019). l-Proline alleviates kidney injury caused by AFB1 and AFM1 through regulating excessive apoptosis of kidney cells. Toxins 11 (4), 226. 10.3390/toxins11040226 30995739 PMC6521284

[B96] Li H. X. Heo M. Go Y. Kim Y. S. Kim Y. H. Yang S. Y. (2020). Coumarin and moracin derivatives from mulberry leaves (*Morus alba* L.) with soluble epoxide hydrolase inhibitory activity. Molecules 25 (17), 3967. 10.3390/molecules25173967 32878149 PMC7504814

[B97] Li H. X. Park J. U. Su X. D. Kim K. T. Kang J. S. Kim Y. R. (2018a). Identification of anti-melanogenesis constituents from *Morus alba* L. Leaves. Mol Basel, Switz. 23 (10), 2559. 10.3390/molecules23102559 PMC6222840 30297610

[B98] Li J. Dou L. Chen S. Zhou H. Mou F. (2021a). Neochlorogenic acid: an anti-HIV active compound identified by screening of Cortex Mori [*Morus Alba* L. (Moraceae)]. Pharm. Biol. 59 (1), 1517–1527. 10.1080/13880209.2021.1995005 34714196 PMC8567877

[B99] Li Y. Zhang X. Liang C. Hu J. Yu Z. (2018b). Safety evaluation of mulberry leaf extract: acute, subacute toxicity and genotoxicity studies. Regul. Toxicol. Pharmacol. 95, 220–226. 10.1016/j.yrtph.2018.03.007 29530616

[B100] Li Z. Chen X. Liu G. Li J. Zhang J. Cao Y. (2021b). Antioxidant activity and mechanism of resveratrol and polydatin isolated from mulberry (*Morus alba* L.). Molecules 26 (24), 7574. 10.3390/molecules26247574 34946655 PMC8709137

[B101] Lim D. W. Jung J. Park J. Baek N. Kim Y. T. Kim I. (2015). Antidepressant-like effects of sanggenon G, isolated from the root bark of *Morus alba*, in rats: involvement of the serotonergic system. Biol. Pharm. Bull. 38 (11), 1772–1778. 10.1248/bpb.b15-00471 26289125

[B102] Lim S. H. Yu J. S. Lee H. S. Choi C. I. Kim K. H. (2021). Antidiabetic flavonoids from fruits of *Morus alba* promoting insulin-stimulated glucose uptake via akt and AMP-activated protein kinase activation in 3T3-L1 adipocytes. Pharmaceutics 13 (4), 526. 10.3390/pharmaceutics13040526 33918969 PMC8069446

[B103] Liu Y. Peng Y. Chen C. Ren H. Zhu J. Deng Y. (2024). Flavonoids from mulberry leaves inhibit fat production and improve fatty acid distribution in adipose tissue in finishing pigs. Anim. Nutr. 16, 147–157. 10.1016/j.aninu.2023.11.003 38357574 PMC10864206

[B104] Liu Y. Zhou X. Zhou D. Jian Y. Jia J. Ge F. (2022). Isolation of chalcomoracin as a potential α-glycosidase inhibitor from mulberry leaves and its binding mechanism. Molecules 27 (18), 5742. 10.3390/molecules27185742 36144478 PMC9504037

[B105] Liu Ying G. M. G. J. (2023). Identification of chemical constituents and optimization of total flavonoids extraction process for Mori Ramulus. Chin. Tradit. Pat. Med. 45 (06), 1892–1901.

[B106] Maqsood M. Anam S. R. Sahar A. Khan M. I. (2022). Mulberry plant as a source of functional food with therapeutic and nutritional applications: a review. J. Food Biochem. 46 (11), e14263. 10.1111/jfbc.14263 35642132

[B107] Martins B. D. A. Sande D. Solares M. D. Takahashi J. A. (2021). Antioxidant role of morusin and mulberrofuran B in ethanol extract of *Morus alba* roots. Nat. Prod. Res. 35 (24), 5993–5996. 10.1080/14786419.2020.1810036 32840147

[B108] Miklasinska M. Kepa M. Wojtyczka R. D. Idzik D. Zdebik A. Orlewska K. (2015). Antibacterial activity of protocatechuic acid ethyl ester on *Staphylococcus aureus* clinical strains alone and in combination with antistaphylococcal drugs. Molecules 20 (8), 13536–13549. 10.3390/molecules200813536 26213908 PMC6332044

[B109] Moon K. M. Yang J. Lee M. Kwon E. Baek J. Hwang T. (2022). Maclurin exhibits antioxidant and anti-tyrosinase activities, suppressing melanogenesis. Antioxidants 11 (6), 1164. 10.3390/antiox11061164 35740060 PMC9220237

[B110] Nomura T. Hano Y. Fukai T. (2009). Chemistry and biosynthesis of isoprenylated flavonoids from Japanese mulberry tree. Proc. Jpn. Acad. Ser. B 85 (9), 391–408. 10.2183/pjab.85.391 19907125 PMC3621561

[B111] Ochiishi T. Kaku M. Kajsongkram T. Thisayakorn K. (2021). Mulberry fruit extract alleviates the intracellular amyloid-β oligomer-induced cognitive disturbance and oxidative stress in Alzheimer's disease model mice. Genes cells. 26 (11), 861–873. 10.1111/gtc.12889 34387016

[B112] Oh K. K. Adnan M. Cho D. H. (2021). Network Pharmacology study on *Morus alba* L. Leaves: pivotal functions of bioactives on RAS signaling pathway and its associated target proteins against gout. Int. J. Mol. Sci. 22 (17), 9372. 10.3390/ijms22179372 34502281 PMC8431517

[B113] Panyatip P. Padumanonda T. Yongram C. Kasikorn T. Sungthong B. Puthongking P. (2022). Impact of tea processing on tryptophan, melatonin, phenolic and flavonoid contents in mulberry (*Morus alba* L.) leaves: quantitative analysis by LC-MS/MS. Molecules 27 (15), 4979. 10.3390/molecules27154979 35956928 PMC9370701

[B114] Park H. J. Park S. H. (2020). Induction of cytoprotective autophagy by morusin via AMP-activated protein kinase activation in human non-small cell lung cancer cells. Nutr. Res. Pract. 14 (5), 478–489. 10.4162/nrp.2020.14.5.478 33029288 PMC7520565

[B115] Park S. H. Kim D. S. Kim S. Lorz L. R. Choi E. Lim H. Y. (2019). Loliolide presents antiapoptosis and antiscratching effects in human keratinocytes. Int. J. Mol. Sci. 20 (3), 651. 10.3390/ijms20030651 30717391 PMC6387290

[B116] Park Y. J. Seong S. H. Kim M. S. Seo S. W. Kim M. R. Kim H. S. (2017). High-throughput detection of antioxidants in mulberry fruit using correlations between high-resolution mass and activity profiles of chromatographic fractions. Plant Methods 13, 108. 10.1186/s13007-017-0258-3 29225663 PMC5718003

[B117] Paudel P. Park S. E. Seong S. H. Jung H. A. Choi J. S. (2019). Novel diels-alder type adducts from *Morus alba* root bark targeting human monoamine oxidase and dopaminergic receptors for the management of neurodegenerative diseases. Int. J. Mol. Sci. 20 (24), 6232. 10.3390/ijms20246232 31835621 PMC6940761

[B118] Peng J. Gong L. Si K. Bai X. Du G. (2011). Fluorescence resonance energy transfer assay for high-throughput screening of ADAMTS1 inhibitors. Molecules 16 (12), 10709–10721. 10.3390/molecules161210709 22186957 PMC6264766

[B119] Phan T. N. Kim O. Ha M. T. Hwangbo C. Min B. S. Lee J. H. (2020). Albanol B from mulberries exerts anti-cancer effect through mitochondria ROS production in lung cancer cells and suppresses *in vivo* tumor growth. Int. J. Mol. Sci. 21 (24), 9502. 10.3390/ijms21249502 33327489 PMC7764986

[B120] Plotnikov M. B. Plotnikova T. M. (2021). Tyrosol as a neuroprotector: strong effects of a "weak" antioxidant. Curr. Neuropharmacol. 19 (4), 434–448. 10.2174/1570159X18666200507082311 32379590 PMC8206466

[B121] Polumackanycz M. Wesolowski M. Viapiana A. (2021). *Morus alba* L. And *Morus nigra* L. Leaves as a promising food source of phenolic compounds with antioxidant activity. Plant Food Hum. Nutr. 76 (4), 458–465. 10.1007/s11130-021-00922-7 PMC8629867 34570290

[B122] Przeor M. (2022). Some common medicinal plants with antidiabetic activity, known and available in europe (A mini-review). Pharmaceuticals 15 (1), 65. 10.3390/ph15010065 35056122 PMC8778315

[B123] Qu Y. Wang L. Guo W. (2019). Screening and identification of antipyretic components in the postfrost leaves of *Morus alba* based on multivariable and continuous-index spectrum-effect correlation. J. Anal. Methods Chem. 2019, 8796276. 10.1155/2019/8796276 31737405 PMC6815998

[B124] Ramos-Romero S. Ponomarenko J. Amezqueta S. Hereu M. Miralles-Perez B. Romeu M. (2022). Fiber-like action of d-fagomine on the gut microbiota and body weight of healthy rats. Nutrients 14 (21), 4656. 10.3390/nu14214656 36364917 PMC9657608

[B125] Ray S. Gupta S. Panda G. Chatterjee P. Das A. Patawri P. (2023). Identification of pseudobaptigenin as a novel polyphenol-based multi-target antagonist of different hormone receptors for breast cancer therapeutics. J. Biomol. Struct. Dyn., 1–13. 10.1080/07391102.2023.2226750 PMC12680423 37409735

[B126] Razliqi R. N. Ahangarpour A. Mard S. A. Khorsandi L. (2023). Gentisic acid ameliorates type 2 diabetes induced by Nicotinamide-Streptozotocin in male mice by attenuating pancreatic oxidative stress and inflammation through modulation of Nrf2 and NF-кB pathways. Life Sci. 325, 121770. 10.1016/j.lfs.2023.121770 37192699

[B127] Ren B. Kwah M. X. Liu C. Ma Z. Shanmugam M. K. Ding L. (2021). Resveratrol for cancer therapy: challenges and future perspectives. Cancer Lett. 515, 63–72. 10.1016/j.canlet.2021.05.001 34052324

[B128] Shaito A. Posadino A. M. Younes N. Hasan H. Halabi S. Alhababi D. (2020). Potential adverse effects of resveratrol: a literature review. Int. J. Mol. Sci. 21 (6), 2084. 10.3390/ijms21062084 32197410 PMC7139620

[B129] Sharma N. Sharma A. Bhatia G. Landi M. Brestic M. Singh B. (2019). Isolation of phytochemicals from Bauhinia variegata L. Bark and their *in vitro* antioxidant and cytotoxic potential. Antioxidants 8 (10), 492. 10.3390/antiox8100492 31627372 PMC6826637

[B130] Shi B. Qian J. Miao H. Zhang S. Hu Y. Liu P. (2023). Mulberroside A ameliorates CCl4-induced liver fibrosis in mice via inhibiting pro-inflammatory response. Food Sci. Nutr. 11 (6), 3433–3441. 10.1002/fsn3.3333 37324833 PMC10261818

[B131] Shrestha S. Seong S. H. Park S. G. Min B. S. Jung H. A. Choi J. S. (2019). Insight into the PTP1B inhibitory activity of arylbenzofurans: an *in vitro* and *in silico* study. Molecules 24 (16), 2893. 10.3390/molecules24162893 31395821 PMC6721227

[B132] Shu Y. Yuan H. Xu M. Hong Y. Gao C. Wu Z. (2021). A novel Diels-Alder adduct of mulberry leaves exerts anticancer effect through autophagy-mediated cell death. Acta Pharmacol. Sin. 42 (5), 780–790. 10.1038/s41401-020-0492-5 32814819 PMC8115316

[B133] Spilioti E. Jaakkola M. Tolonen T. Lipponen M. Virtanen V. Chinou I. (2014). Phenolic acid composition, antiatherogenic and anticancer potential of honeys derived from various regions in Greece. PLoS One 9 (4), e94860. 10.1371/journal.pone.0094860 24752205 PMC3994057

[B134] Su M. Cui J. Zhao J. Fu X. (2023). Skimmin ameliorates cardiac function via the regulation of M2 macrophages in a myocardial infarction mouse model. Perfusion-UK 38 (6), 1298–1307. 10.1177/02676591221100742 35532100

[B135] Suriyaprom S. Kaewkod T. Promputtha I. Desvaux M. Tragoolpua Y. (2021). Evaluation of antioxidant and antibacterial activities of white mulberry (*Morus alba* L.) fruit extracts. Plants 10 (12), 2736. 10.3390/plants10122736 34961207 PMC8703457

[B136] Sutanto C. N. Loh W. W. Kim J. E. (2022). The impact of tryptophan supplementation on sleep quality: a systematic review, meta-analysis, and meta-regression. Nutr. Rev. 80 (2), 306–316. 10.1093/nutrit/nuab027 33942088

[B137] Suthamwong P. Minami M. Okada T. Shiwaku N. Uesugi M. Yokode M. (2020). Administration of mulberry leaves maintains pancreatic β-cell mass in obese/type 2 diabetes mellitus mouse model. BMC Complement. Med. Ther. 20 (1), 136. 10.1186/s12906-020-02933-4 32375753 PMC7201661

[B138] Tang W. H. Zhang Z. N. Cai H. R. Sun W. Yang H. Zhao E. H. (2023). Effect of *Morus alba* extract sanggenon C on growth and proliferation of glioblastoma cells. Zhongguo Zhong Yao Za Zhi 48 (1), 211–219. 10.19540/j.cnki.cjcmm.20220905.701 36725273

[B139] Thomas P. S. Eilstein J. Prasad A. Ekhar P. Shetty S. Peng Z. (2022). Comprehensive characterization of naturally occurring antioxidants from the twigs of mulberry (*Morus alba*) using on-line high-performance liquid chromatography coupled with chemical detection and high-resolution mass spectrometry. Phytochem. Anal. 33 (1), 105–114. 10.1002/pca.3072 34184340 PMC9292295

[B140] Tianqiao Yong D. L. C. X. Liang D. Xiao C. Huang L. Chen S. Xie Y. (2022). Hypouricemic effect of 2,4-dihydroxybenzoic acid methyl ester in hyperuricemic mice through inhibiting XOD and down-regulating URAT1. Biomed. Pharmacother. 153, 113303. 10.1016/j.biopha.2022.113303 35750011

[B141] Wang M. Lin L. Lu J. Chen X. (2021). Pharmacological review of isobavachalcone, a naturally occurring chalcone. Pharmacol. Res. 165, 105483. 10.1016/j.phrs.2021.105483 33577976

[B142] Wang Q. Zhang L. Pang P. (2023a). Dihydrokaempferol attenuates LPS-induced inflammation and apoptosis in WI-38 cells. Allergol. Immunopath. 51 (6), 23–29. 10.15586/aei.v51i6.971 37937492

[B143] Wang W. Mei L. Yue H. Tao Y. Liu Z. (2023b). Targeted isolation of cyclooxygenase-2 inhibitors from Saussurea obvallata using affinity ultrafiltration combined with preparative liquid chromatography. J. Chromatogr. B 1217, 123620. 10.1016/j.jchromb.2023.123620 36773385

[B144] Wang Y. Xiang L. Wang C. Tang C. He X. (2013). Antidiabetic and antioxidant effects and phytochemicals of mulberry fruit (*Morus alba* L.) polyphenol enhanced extract. PLoS One 8 (7), e71144. 10.1371/journal.pone.0071144 23936259 PMC3728024

[B145] Wang Z. Cui J. Li D. Ran S. Huang J. Chen G. (2023c). Morin exhibits a neuroprotective effect in MPTP-induced Parkinson's disease model via TFEB/AMPK-mediated mitophagy. Phytomedicine 116, 154866. 10.1016/j.phymed.2023.154866 37209604

[B146] Wasilewicz A. Kirchweger B. Bojkova D. Abi Saad M. J. Langeder J. Bütikofer M. (2023). Identification of natural products inhibiting SARS-CoV-2 by targeting viral proteases: a combined *in silico* and *in vitro* approach. J. Nat. Prod. 86 (2), 264–275. 10.1021/acs.jnatprod.2c00843 36651644 PMC9885530

[B147] Wei T. Ji X. Xue J. Gao Y. Zhu X. Xiao G. (2021). Cyanidin-3-O-glucoside represses tumor growth and invasion *in vivo* by suppressing autophagy via inhibition of the JNK signaling pathways. Food Funct. 12 (1), 387–396. 10.1039/d0fo02107e 33326533

[B148] Wenmin Du Z. Z. J. W. (2022). Herbal textual research on mori in famous classical formulas. Chin. J. Exp. Traditional Med. Formulae 28 (10), 11–21. 10.13422/j.cnki.syfjx.20212149

[B149] Won Y. Seo K. (2020). Sanggenol L induces apoptosis and cell cycle arrest via activation of p53 and suppression of PI3K/Akt/mTOR signaling in human prostate cancer cells. Nutrients 12 (2), 488. 10.3390/nu12020488 32075054 PMC7071324

[B150] Wu H. E. Su C. C. Wang S. C. Liu P. L. Cheng W. C. Yeh H. C. (2023). Anticancer effects of morusin in prostate cancer via inhibition of akt/mTOR signaling pathway. Am. J. Chin. Med. 51 (4), 1019–1039. 10.1142/S0192415X23500477 37120705

[B151] Xu L. Yu M. Niu L. Huang C. Wang Y. Wu C. (2020). Phenolic compounds isolated from *Morus nigra* and their alpha-glucosidase inhibitory activities. Nat. Prod. Res. 34 (5), 605–612. 10.1080/14786419.2018.1491041 30369248

[B152] Yang J. Chen X. Rao S. Li Y. Zang Y. Zhu B. (2022a). Identification and quantification of flavonoids in okra (abelmoschus esculentus L. Moench) and antiproliferative activity *in vitro* of four main components identified. Metabolites 12 (6), 483. 10.3390/metabo12060483 35736417 PMC9228595

[B153] Yang Q. Kang Z. Zhang J. Qu F. Song B. (2021). Neuroprotective effects of isoquercetin: an *in vitro* and *in vivo* study. Cell J. (Yakhteh) 23 (3), 355–365. 10.22074/cellj.2021.7116 PMC8286454 34308580

[B154] Yang T. Y. Wu Y. L. Yu M. H. Hung T. W. Chan K. C. Wang C. J. (2022b). Mulberry leaf and neochlorogenic acid alleviates glucolipotoxicity-induced oxidative stress and inhibits proliferation/migration via downregulating ras and FAK signaling pathway in vascular smooth muscle cell. Nutrients 14 (15), 3006. 10.3390/nu14153006 35893859 PMC9331252

[B155] Yang Y. Gong T. Liu C. Chen R. Chinese A. O. M. S. Key L. O. B. S. (2010). Four new 2-arylbenzofuran derivatives from leaves of *Morus alba* L. Chem. Pharm. Bull. 58 (2), 257–260. 10.1248/cpb.58.257 20118592

[B156] Yang Z. Matsuzaki K. Takamatsu S. Kitanaka S. (2011). Inhibitory effects of constituents from *Morus alba* var. multicaulis on differentiation of 3T3-L1 cells and nitric oxide production in RAW264.7 cells. Molecules 16 (7), 6010–6022. 10.3390/molecules16076010 21772233 PMC6264761

[B157] Yao J. He H. Xue J. Wang J. Jin H. Wu J. (2019). Mori ramulus (Chin.Ph.)-the dried twigs of *Morus alba* L./Part 1: discovery of two novel coumarin glycosides from the anti-hyperuricemic ethanol extract. Molecules 24 (3), 629. 10.3390/molecules24030629 30754654 PMC6384676

[B158] Yu J. S. Lim S. H. Lee S. R. Choi C. Kim K. H. (2021). Antioxidant and anti-inflammatory effects of white mulberry (*Morus alba* L.) fruits on lipopolysaccharide-stimulated RAW 264.7 macrophages. Molecules 26 (4), 920. 10.3390/molecules26040920 33572374 PMC7916181

[B159] Zabady S. Mahran N. Soltan M. A. Alaa E. M. Eid R. A. Albogami S. (2022). Cyanidin-3-Glucoside modulates hsa_circ_0001345/miRNA106b/atg16l1 Axis expression as a potential protective mechanism against hepatocellular carcinoma. Curr. Issues Mol. Biol. 44 (4), 1677–1687. 10.3390/cimb44040115 35723373 PMC9164082

[B160] Zeng Q. Chen M. Wang S. Xu X. Li T. Xiang Z. (2022). Comparative and phylogenetic analyses of the chloroplast genome reveal the taxonomy of the *Morus* genus. Front. Plant Sci. 13, 1047592. 10.3389/fpls.2022.1047592 36507423 PMC9729782

[B161] Zhang L. Tao G. Chen J. Zheng Z. (2016). Characterization of a new flavone and tyrosinase inhibition constituents from the twigs of *Morus alba* L. Molecules 21 (9), 1130. 10.3390/molecules21091130 27598113 PMC6274457

[B162] Zhao D. Shi Y. Petrova V. Yue G. G. L. Negrin A. Wu S. (2019). Jaboticabin and related polyphenols from jaboticaba (myrciaria cauliflora) with anti-inflammatory activity for chronic obstructive pulmonary disease. J. Agric. Food Chem. 67 (5), 1513–1520. 10.1021/acs.jafc.8b05814 30675793

[B163] Zhao X. Fu Z. Yao M. Cao Y. Zhu T. Mao R. (2022). Mulberry (*Morus alba* L.) leaf polysaccharide ameliorates insulin resistance- and adipose deposition-associated gut microbiota and lipid metabolites in high-fat diet-induced obese mice. Food Sci. Nutr. 10 (2), 617–630. 10.1002/fsn3.2689 35154697 PMC8825736

[B164] Zheng Y. Lee E. H. Lee S. Y. Lee Y. Shin K. O. Park K. (2023). *Morus alba* L. root decreases melanin synthesis via sphingosine-1-phosphate signaling in B16F10 cells. J. Ethnopharmacol. 301, 115848. 10.1016/j.jep.2022.115848 36272492

[B165] Zhou H. Yan X. Yu W. Liang X. Du X. Liu Z. (2022a). Lactic acid in macrophage polarization: the significant role in inflammation and cancer. Int. Rev. Immunol. 41 (1), 4–18. 10.1080/08830185.2021.1955876 34304685

[B166] Zhou J. Li S. X. Wang W. Guo X. Y. Lu X. Y. Yan X. P. (2013). Variations in the levels of mulberroside A, oxyresveratrol, and resveratrol in mulberries in different seasons and during growth. Sci. World J. 2013, 380692. 10.1155/2013/380692 PMC3760103 24023529

[B167] Zhou Q. Y. Liao X. Kuang H. Li J. Zhang S. (2022b). LC-MS metabolite profiling and the hypoglycemic activity of *Morus alba* L. Extracts. Molecules 27 (17), 5360. 10.3390/molecules27175360 36080128 PMC9457631

[B168] Zhu M. Tang X. Zhu Z. Gong Z. Tang W. Hu Y. (2023). STING activation in macrophages by vanillic acid exhibits antineoplastic potential. Biochem. Pharmacol. 213, 115618. 10.1016/j.bcp.2023.115618 37211172

