# Supplementary material for: Comprehensive overview of different medicinal parts from Morus alba L.: chemical compositions and pharmacological activities
Source: Front Pharmacol. 2024 Apr 17;15:1364948. doi: 10.3389/fphar.2024.1364948 (PMC11061381; doi:10.3389/fphar.2024.1364948)
Supplement: Supplementary file 1 [file Table1.pdf]

Table S1 Phenols in *Morus alba* L.

No.	Name	Source	Pharmacological property	Reference
1	albanol B	C	anti-cancer	(Paudel et al., 2019) (Phan et al., 2020)
2	apigenin	C	anti-inflammatory; anti-cancer	(Kavitha and Geetha, 2018)
3	astragalin	A	antioxidant; anti-inflammatory	(Kim et al., 2020) (Yu et al., 2021)
4	biochanin A	C	anti-inflammatory; antidiabetic; anti-cancer; lipid metabolism regulation	(Kavitha and Geetha, 2018)
5	chrysin	C	anti-cancer	(Kavitha and Geetha, 2018) (Alavi et al., 2023)
6	cudraflavone B	C	anti-cancer; anti-inflammatory	(Kollar et al., 2013)
7	cyclomulberrin	D	anti-cancer	(Zhang et al., 2016) (Alibakhshi et al., 2023)
8	dihydromorin	D	antidiabetic	(Kwon et al., 2022)
9	dihydrokaempferol	D	anti-inflammatory	(Zhang et al., 2016) (Wang et al., 2023)
10	dihydrokaempferol 7-O-β-D-glucopyranoside	B	antidiabetic; antioxidant	(Wang et al., 2013)
11	eriodictyol	D	antioxidant; anti-inflammatory; anti-cancer; neuroprotection; heart protection; anti-diabetes; anti-obesity; liver protection	(Zhang et al., 2016) (Islam et al., 2020)
12	genistein	C	anti-inflammatory	(Kavitha and Geetha, 2018)
13	isoquercetin	A; B; D	vascular protection; antioxidant; neuroprotection	(Kim et al., 2017) (Kim et al., 2014) (Liu Ying, 2023) (Yang et al., 2021)
14	isorhamnetin	A	anti-melanogenesis	(Jeong et al., 2015)
15	kaempferol	A; D	antioxidant; anti-Inflammatory	(Jeong et al., 2015) (Liu Ying, 2023)
16	kaempferol 3-O-rhamnoside-7-O-glucoside	A	—	(Ju et al., 2018)

17	kaempferol 3-O-rutinoside-7-O-glucoside	A	—	(Ju et al., 2018)
18	kaempferol 3-O-β-D-rutinoside	B	renal protection	(Wang et al., 2013) (Lee et al., 2018)
19	kaempferol-3,7-di-O-glucoside	A	—	(Kim et al., 2020)
20	kaempferol-3-O-rutinoside	B	cardiac protection	(D'Urso et al., 2019) (Hua et al., 2022)
21	kuwanon A	C	anti-inflammatory	(Yang et al., 2011) (Baek et al., 2021)
22	kuwanon B	C	antibiosis	(Baek et al., 2021) (Dong et al., 2023)
23	kuwanon C	C; D	anti-inflammatory; antidiabetic	(Jeong et al., 2015) (Baek et al., 2021) (Kwon et al., 2022)
24	kuwanon T	C	anti-inflammatory	(Yang et al., 2011)
25	luteolin	A	anti-inflammatory; antioxidant	(Park et al., 2017) (Yu et al., 2021)
26	morin	A	anti-inflammatory; antidiabetic	(Kavitha and Geetha, 2018) (Przeor, 2022)
27	morin hydrate	C	antiviral	(Hong et al., 2020)
28	morusin	C; D	antidiabetic; anti-melanogenesis	(Kim et al., 2020) (Chaita et al., 2017)
29	morusinol	C	antidiabetic; anticancer	(Guo et al., 2023)
30	morusone	D	—	(Zhang et al., 2016)
31	myricetin	A	antioxidant	(Polumackanycz et al., 2021)
32	naringenin	A	antioxidant	(Polumackanycz et al., 2021)
33	norartocarpetin	A; D	antidiabetic	(Jeong et al., 2015) (Kwon et al., 2022)
34	pseudobaptigenin	C	anti-inflammatory; anticancer	(Kavitha and Geetha, 2018) (Ray et al., 2023)
35	quercetin	A; B; D	antioxidant; anti-Inflammatory	(Jeong et al., 2015) (Liu Ying, 2023) (Yu et al., 2021)
36	quercetin 3,7-di-O-β-D-glucopyranoside	B	antidiabetic; antioxidant	(Wang et al., 2013)
37	quercetin	B	antidiabetic; antioxidant	(Wang et al., 2013)

	3-O-(6"-O-acetyl)-β-D-glucopyranoside			
38	quercetin 3-O-rutinoside-7-O-glucoside	B	—	(D'Urso et al., 2019)
39	quercetin 3-O-β-D-rutinoside	B	antidiabetic; antioxidant	(Wang et al., 2013)
40	quercetin 7-O-β-D-glucopyranoside	B	antidiabetic; antioxidant	(Wang et al., 2013)
41	quercetin-3,7-di-O-glucoside	A	—	(Kim et al., 2020)
42	quercetin-3-gentiobioside	A	anticancer	(Kim et al., 2020) (Yang et al., 2022)
43	quercitrin	A	antioxidant; anti-inflammatory; antimicrobial; immunomodulatory; analgesic	(Kim et al., 2020) (Chen et al., 2022)
44	rutin	B	antioxidant; antidiabetic	(Chen et al., 2022) (Hunyadi et al., 2012)
45	sanggenol A	C	anti-bacteria; antiviral	(Ko et al., 2021) (Chen et al., 2022) (Grienke et al., 2016)
46	sanggenol L	C	anticancer	(Won and Seo, 2020)
47	sanggenon A	C	anti-inflammatory	(Ko et al., 2021)
48	sanggenon C	C	Cardioprotection; anticancer; antiviral	(Wasilewicz et al., 2023) (Gu et al., 2017) (Tang et al., 2023) (Wasilewicz et al., 2023)
49	sanggenon F	C	anti-obesity	(Yang et al., 2011)
50	sanggenon G	C	anti-depression; antiviral	(Lim et al., 2015) (Wasilewicz et al., 2023)
51	sanggenon M	C; D	anti-inflammatory; antidiabetic	(Ko et al., 2021) (Liu Ying, 2023) (Xu et al., 2020)
52	sanggenon O	C	antiviral	(Wasilewicz et al., 2023)

53	steppogenin	A; C; D	anticancer	(Jeong et al., 2015) (Cha et al., 2023)
54	taxifolin	A; D	antioxidant; anti-inflammatory	(Kim et al., 2020) (Liu Ying, 2023)
55	vitexin	C	anti-inflammatory; anticancer; neuroprotection	(Kavitha and Geetha, 2018)
56	5,7-dihydroxyisoflavone	C	anti-inflammatory; anticancer, neuroprotection	(Kavitha and Geetha, 2018)
57	5,7-dimethoxy flavone	C	anti-inflammatory; anticancer; neuroprotection	(Kavitha and Geetha, 2018)
58	5,7,2′,4′-tetrahydroxy-3-methoxyflavone	D	—	(Zhang et al., 2016)
59	5,7,3′-trihydroxy-flavanone-4′-O-β-D-glucopyranoside	B	—	(Wang et al., 2013)
60	5,7,4′-trihydroxy-flavanone-3′-O-β-D-glucopyranoside	B	—	(Wang et al., 2013)
61	albafuran C	C	—	(Peng et al., 2011)
62	benzyl D-glucopyranoside	A	—	(Doi et al., 2001)
63	caffeic acid	A	antioxidant	(Panyatip et al., 2022) (Park et al., 2017) (Polumackanycz et al., 2021)
64	chlorogenic acid	A; B; C; D	antioxidant	(Kim et al., 2020) (Chen et al., 2022) (Liu Ying, 2023)
65	curcumin	C	anti-inflammatory; anticancer	(Kavitha and Geetha, 2018)
66	dihydroconiferyl alcohol	B	anti-inflammatory	(Lee et al., 2021)
67	dihydrooxyresveratrol	D	anti-inflammatory; anti-melanogenesis	(Chaita et al., 2017) (Jongkon et al., 2022)
68	eugenol	C	anti-inflammatory; anticancer	(Kavitha and Geetha, 2018)
69	ferulic acid	A	antioxidant	(Polumackanycz et al., 2021)
70	gallic acid	A	antioxidant	(Panyatip et al., 2022)

				(Park et al., 2017)<br>(Polumackanycz et al., 2021)
71	gastrodin	A	antioxidant	(Ganzon et al., 2018)
72	gentisic acid	A	antidiabetic	(Panyatip et al., 2022)<br>(Park et al., 2017)<br>(Razliqi et al., 2023)
73	jaboticabin	B	anti-inflammatory	(Wang et al., 2013)<br>(Zhao et al., 2019)
74	kuwanon E	C	anti-inflammatory; anticancer	(Kollar et al., 2013)
75	kuwanon G	C; D	antidiabetic;<br>anti-neurodegenerative	(Kim et al., 2020)<br>(Paudel et al., 2019)<br>(Kwon et al., 2022)
76	kuwanon H	C	anti-melanogenesis;<br>antidiabetic	(Chaita et al., 2017)<br>(Zhou et al., 2022)
77	kuwanon J	C	anti-melanogenesis	(Nomura et al., 2009)<br>(Hu et al., 2018)
78	maclurin	D	antioxidant; anti-cancer;<br>against Benzo[a]pyrene	(Moon et al., 2022)<br>(Lee and Lee, 2021)
79	methyl benzoate	B	anti-inflammatory	(Lee et al., 2021)
80	moracin B	A; D	antidiabetic;<br>anti-inflammatory	(Jeon and Choi, 2019)
81	moracin C	A	antidiabetic; anti-bacteria	(Jeon and Choi, 2019)<br>(Kim et al., 2012)
82	moracin D	D	anticancer; anti-obesity	(Zhang et al., 2016)<br>(Yang et al., 2011)
83	moracin J	A; D	anti-melanogenesis;<br>anti-inflammatory	(Zhang et al., 2016)<br>(Li et al., 2018)<br>(Li et al., 2020)
84	moracin M	A; D	anti-melanogenesis;<br>antidiabetic;<br>anti-inflammatory	(Chaita et al., 2017)<br>(Jeon and Choi, 2019)<br>(Li et al., 2020)
85	moracin M 3'-O-β-glucopyranoside	A	anti-inflammatory;<br>anti-melanogenesis	(Li et al., 2020)<br>(Li et al., 2018)
86	moracin M 6-O-β-D-glucopyranoside	A	anti-melanogenesis	(Li et al., 2018)
87	moracin N	A	antidiabetic; anticancer	(Jeon and Choi, 2019)<br>(Gao et al., 2020)
88	moracin O	C	anti-inflammatory;<br>anti-obesity; anticancer	(Yang et al., 2011)<br>(Ko et al., 2021)<br>(Gao et al., 2020)

89	moracin P	C	anti-inflammatory;<br>anti-obesity	(Yang et al., 2011)<br>(Hardianti et al., 2020)
90	moracin R	C	anti-obesity	(Yang et al., 2011)
91	moracin V	A	__	(Yang et al., 2010)
92	moracin W	A	__	(Yang et al., 2010)
93	moracin X	A	anti-melanogenesis	(Jeong et al., 2015)<br>(Yang et al., 2010)
94	moracin Y	A	anticancer	(Yang et al., 2010)
95	morunigrol C	A	anti-melanogenesis	(Jeong et al., 2015)
96	morusalfuran B	C	anti-melanogenesis	(Shrestha et al., 2019)
97	mulberrofuran B	C	anti-inflammatory;<br>antioxidant	(Ko et al., 2021)<br>(Martins et al., 2021)
98	mulberrofuran D	C; D	anti-melanogenesis	(Shrestha et al., 2019)<br>(Liu Ying, 2023)
99	mulberrofuran D2	C	anti-melanogenesis	(Shrestha et al., 2019)
100	mulberrofuran G	C	anti-neurodegenerative;<br>anti-inflammatory	(Paudel et al., 2019)<br>(Ko et al., 2021)
101	mulberrofuran H	C	anti-melanogenesis	(Shrestha et al., 2019)
102	mulberrofuran J	C	__	(Peng et al., 2011)
103	mulberrofuran L	C	anti-obesity	(Yang et al., 2011)
104	mulberrofuran Y	C	anti-obesity	(Yang et al., 2011)
105	mulberroside A	C; D	antioxidant	(Kim et al., 2020)<br>(Thomas et al., 2022)
106	mulberroside C	C	antiplatelet	(Kwon et al., 2021)
107	mulberroside F	A	anti-inflammatory;<br>anti-melanogenesis	(Lee et al., 2002)<br>(Li et al., 2020)
108	neochlorogenic acid	A	anticancer;<br>anti-inflammatory; antiviral	(Qu et al., 2019)<br>(Yang et al., 2022)<br>(Gao et al., 2020)<br>(Li et al., 2021)
109	oxyresveratrol	C; D	antidiabetic;<br>anti-inflammatory;<br>anti-melanogenesis	(Chaita et al., 2017)<br>(Chen et al., 2013)<br>(Kwon et al., 2022)
110	oxyresveratrol 2-O-β-D-glucopyranoside	C; D	anti-obesity	(Yang et al., 2011)
111	oxyresveratrol 3'-O-β-D-glucoside	D	__	(Choi et al., 2013)
112	p-coumaric acid	A; D	antioxidant	(Zhang et al., 2016)<br>(Polumackanycz et al.,

				2021)
113	p-hydroxybenzoic acid	B	Antidiabetic; antioxidant; anticancer	(Wang et al., 2013) (Spilioti et al., 2014)
114	polydatin	C	antioxidant; anti-hyperuricemia	(Li et al., 2021) (Ge et al., 2023)
115	protocatechuic acid	A	antioxidant; anti-inflammatory; anti-obesity	(Panyatip et al., 2022) (Leyva-Jimenez et al., 2020)
116	protocatechuic acid ethyl ester	B	antidiabetic; antioxidant; antibacterial	(Wang et al., 2013) (Miklasinska et al., 2015)
117	protocatechuic acid methyl ester	B	antidiabetic; antioxidant	(Wang et al., 2013) (Sharma et al., 2019)
118	pyrocatechol	B	antidiabetic; antioxidant; anti-inflammatory	(Wang et al., 2013) (Funakoshi-Tago et al., 2020)
119	resorcinol	D	anti-melanogenesis; antioxidant,	(Chaita et al., 2017) (Agus et al., 2022)
120	resveratrol	B; C; D	antioxidant; anticancer	(Zhou et al., 2013) (Li et al., 2021) (Ren et al., 2021)
121	rhein	C	anti-inflammatory	(Kavitha and Geetha, 2018)
122	rosmarinic acid	A	antioxidant	(Polumackanycz et al., 2021)
123	sanggenofuran A	C	anti-melanogenesis	(Shrestha et al., 2019)
124	sinapinic acid	A	antioxidant	(Polumackanycz et al., 2021)
125	syringic acid	A	antioxidant; antidiabetic	(Jan et al., 2022)
126	threo-guaiacylglycerol-β-O-4′-dihydroconiferyl alcohol	B	—	(Lee et al., 2021)
127	t-resveratrol	D	vascular protection	(Choi et al., 2013) (Guzman et al., 2018)
128	tyrosol	B	antidiabetic; antioxidant; neuroprotection; anti-inflammatory	(Wang et al., 2013) (Plotnikov and Plotnikova, 2021)
129	vanillic acid	B	antidiabetic; antioxidant; anticancer	(Wang et al., 2013) (Zhu et al., 2023)
130	2-(3,5-dihydroxyphenyl)-5,6-dihydroxybenzofuran	A	anti-melanogenesis	(Jeong et al., 2015)

131	2,4-dihydroxybenz aldehyde	D	anti-melanogenesis	(Chaita et al., 2017)
132	2,4-dihydroxybenz oic acid	D	hypouricemic	(Zhang et al., 2016) (Tianqiao Yong, 2022)
133	2-phenylethyl d-rutinoside	B	kidney protection	(Lee et al., 2018)
134	3′,5′-dihydroxy-6-methoxy-7-prenyl-2-arylbenzofuran	C	anti-obesity	(Yang et al., 2011)
135	3-hydroxy-1-(4-hy droxyphenyl)-1-pr opanone	B	anti-inflammatory	(Lee et al., 2021)
136	4-O-caffeoylquinic acid	A	antioxidant	(Qu et al., 2019) (Ganzon et al., 2018)
137	4-hydroxyphenylac etic acid methyl ester	B	—	(Wang et al., 2013)
138	4'-O-methylkuwan on E	C	anticancer	(Kollar et al., 2013)
139	4'-prenyloxyresver atrol	A	anti-melanogenesis	(Jeon and Choi, 2019)
140	5,7-dihydroxychro mone	B	antidiabetic; antioxidant; neuroprotection	(Wang et al., 2013) (Kim et al., 2015)

Note: A: Mori folium; B: Mori fructus; C:Mori cortex; D: Mori ramulus
